# Effects of a synbiotic on the fecal microbiome and metabolomic profiles of healthy research cats administered clindamycin: a randomized, controlled trial

**DOI:** 10.1080/19490976.2018.1560754

**Published:** 2019-02-01

**Authors:** Jacqueline C. Whittemore, Jennifer E. Stokes, Joshua M. Price, Jan S. Suchodolski

**Affiliations:** a The Department of Small Animal Clinical Sciences, University of Tennessee College of Veterinary Medicine, Knoxville, TN, USA; b The Office of Information Technology, University of Tennessee College of Veterinary Medicine, Knoxville, TN, USA; c The Gastrointestinal Laboratory, Small Animal Clinical Sciences, Texas A&M University, TX, USA

**Keywords:** Antibiotic-associated, gastrointestinal signs, antibiotic-associated diarrhea, dysbiosis, short-chain fatty acid, bile acid, indole, polyamine, sphingolipid, cinnaminic acid, probiotic

## Abstract

Reduction in antibiotic-associated gastrointestinal signs (AAGS) in people co-administered probiotics is believed to result from shifts in the microbiome and metabolome. Amelioration of AAGS in cats secondary to synbiotic administration has recently been demonstrated. Thus, the aim of this randomized, double-blinded, placebo-controlled trial was to characterize associated changes in the fecal microbiome and metabolome. Sixteen healthy research cats received clindamycin with food, followed 1 h later by either a placebo or synbiotic, daily for 21 days. Fecal samples were collected during baseline, antibiotic administration, and 6 weeks after antibiotic discontinuation. Sequencing of 16S rRNA genes was performed, and mass spectrometry was used to determine fecal metabolomic profiles. Results were compared using mixed-model analyses, with *P* < 0.05 considered significant. Alpha and beta diversity were altered significantly during treatment, with persistent changes in the Shannon and dysbiosis indices. The relative abundance of *Actinobacteria* (*Adlercreutzia, Bifidobacterium, Collinsella, Slackia*), *Bacteroidia* (*Bacteroides, Prevotella*), *Ruminococcaceae* (*Faecalibacterium, Ruminococcus*), *Veillonellaceae* (*Megamonas, Megasphaera, Phascolarctobacterium*) and *Erysipelotrichaceae* ([*Eubacterium*]) decreased and relative abundance of *Clostridiaceae* (*Clostridium*) and *Proteobacteria* (*Enterobacteriaceae*) increased during treatment, followed by variable return to baseline relative abundances. Derangements in short-chain fatty acid (SCFA), bile acid, tryptophan, sphingolipid, polyamine, benzoic acid, and cinnaminic acid pathways occurred with significant group by time, group, and time interactions for 10, 5, and 106 metabolites, respectively. Of particular note were changes related to polyamine synthesis. Further investigation is warranted to elucidate the role of these alterations in prevention of AAGS in cats, people, and other animals treated with synbiotics.

## Introduction

Clindamycin and other antibiotics can cause antibiotic-associated gastrointestinal signs (AAGS) in cats^1–3^ and people.^4^ The postulated mechanism for the development of AAGS is derangement of the intestinal microbiome, which has been shown to persist up to 4 years after short-term antibiotic administration in people.^5^ Antibiotic administration has been associated with alterations in metabolites with roles in immune function, maintaining enterocyte tight junctions, and glucose homeostasis.^1,6^ Significant differences were found in the fecal microbiome and metabolome of healthy research cats co-administered high dose clindamycin and a placebo or a synbiotic (Proviable-*DC*®, Nutramax Laboratories Veterinary Sciences, Inc., Lancaster, SC USA) once daily for 21 days in one randomized trial, although AAGS did not differ between groups.^1^ In a follow-up trial,^2^ cats were administered a lower dose of clindamycin, followed 1 h later by either a placebo or higher dose of the same synbiotic. Cats receiving the synbiotic had significantly higher food intake, vomited less, and were more likely to complete the initial phase of treatment. Based on these results, we hypothesized that antibiotic-induced changes in the fecal microbiome and metabolomic profiles would differ between treatment groups.

The primary purpose of this study was to characterize changes in the fecal microbiome and metabolomic profiles of healthy research cats administered 75 mg clindamycin PO once daily, followed 1 h later by either two capsules of a placebo or a synbiotic in a randomized double-blinded placebo-controlled trial. The secondary aim was to compare changes with those found in a previously reported study^1^ in which clindamycin and the synbiotic were co-administered.

## Results

### Study population

The placebo group included five female spayed and three male castrated cats, with a median age of 7 years (range 5–10 years) and a median weight of 3.9 kg (range 3.3–6.2 kg). Four cats were withdrawn from treatment due to marked AAGS (vomiting on 3 consecutive days with or without hematemesis). As a result, fecal samples were available for eight cats from this group during baseline (days 5–7) and recovery (days 68–70) but only for four cats at the conclusion of antibiotic administration (days 26–28). Cats in the synbiotic group were comprised of two female spayed and six male castrated cats, with a median age of 9 years (range 5–10 years) and median weight of 4.3 kg (3.4–5.3 kg). All cats that received the synbiotic completed the treatment period, resulting in samples from eight cats at each time point.

### Fecal microbiome

**Figure 1.**
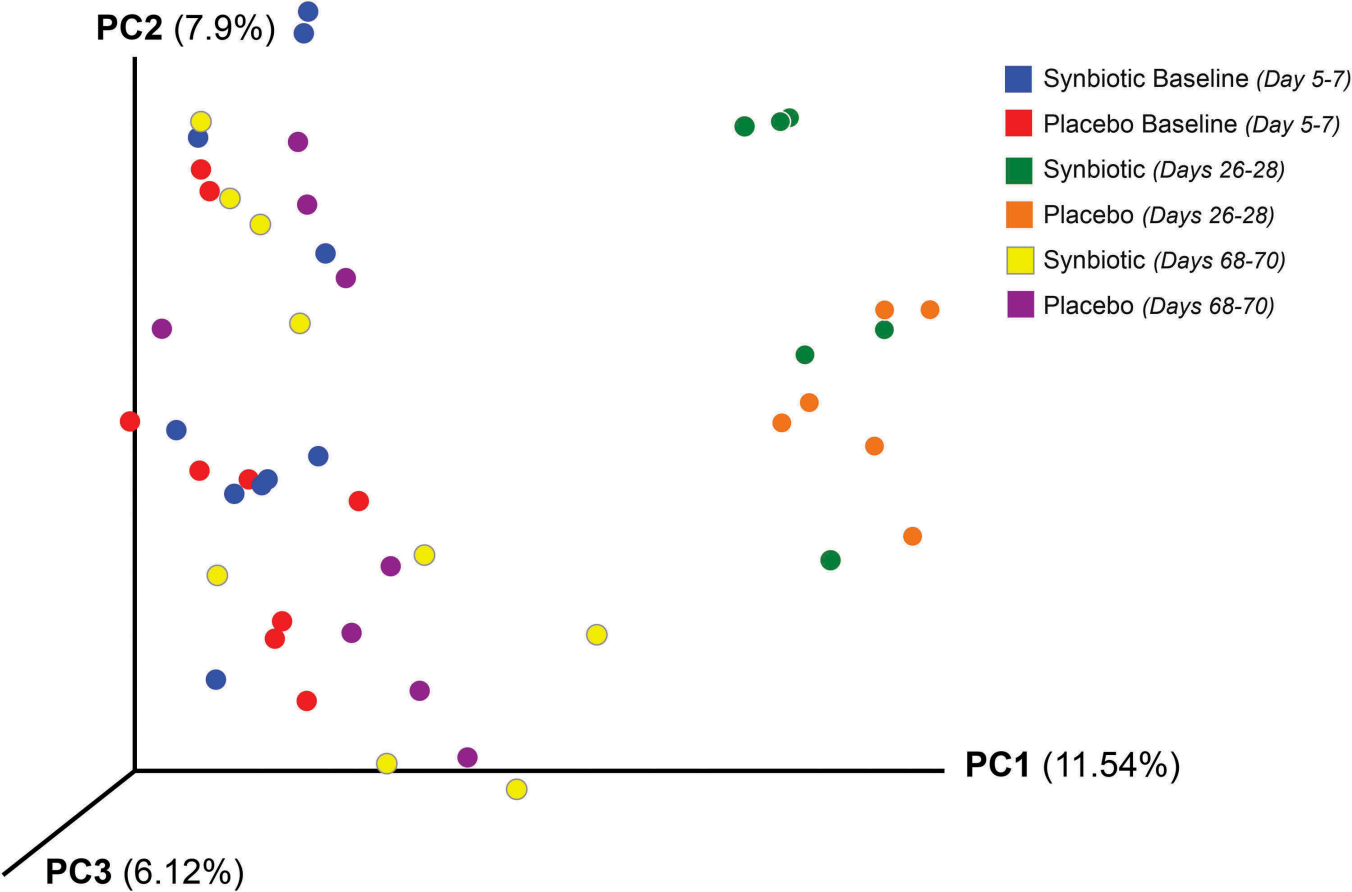
Principal coordinate analysis (PCoA) of unweighted uniFrac distances of 16S rRNA genes. Gene sequences were determined using fecal samples collected at baseline (days 5–7), at the conclusion of antibiotic administration (days 26–28), and after a 6-week washout (days 68–70) from 16 healthy cats, 8 per group,^+^ that received 75 mg clindamycin followed 1 h later by either two capsules of a placebo or synbiotic PO once daily for 21 days. ^+^Feces not available from four cats at time point 26–28 because of early termination of treatment due to severe gastrointestinal signs.

**Figure 2.**
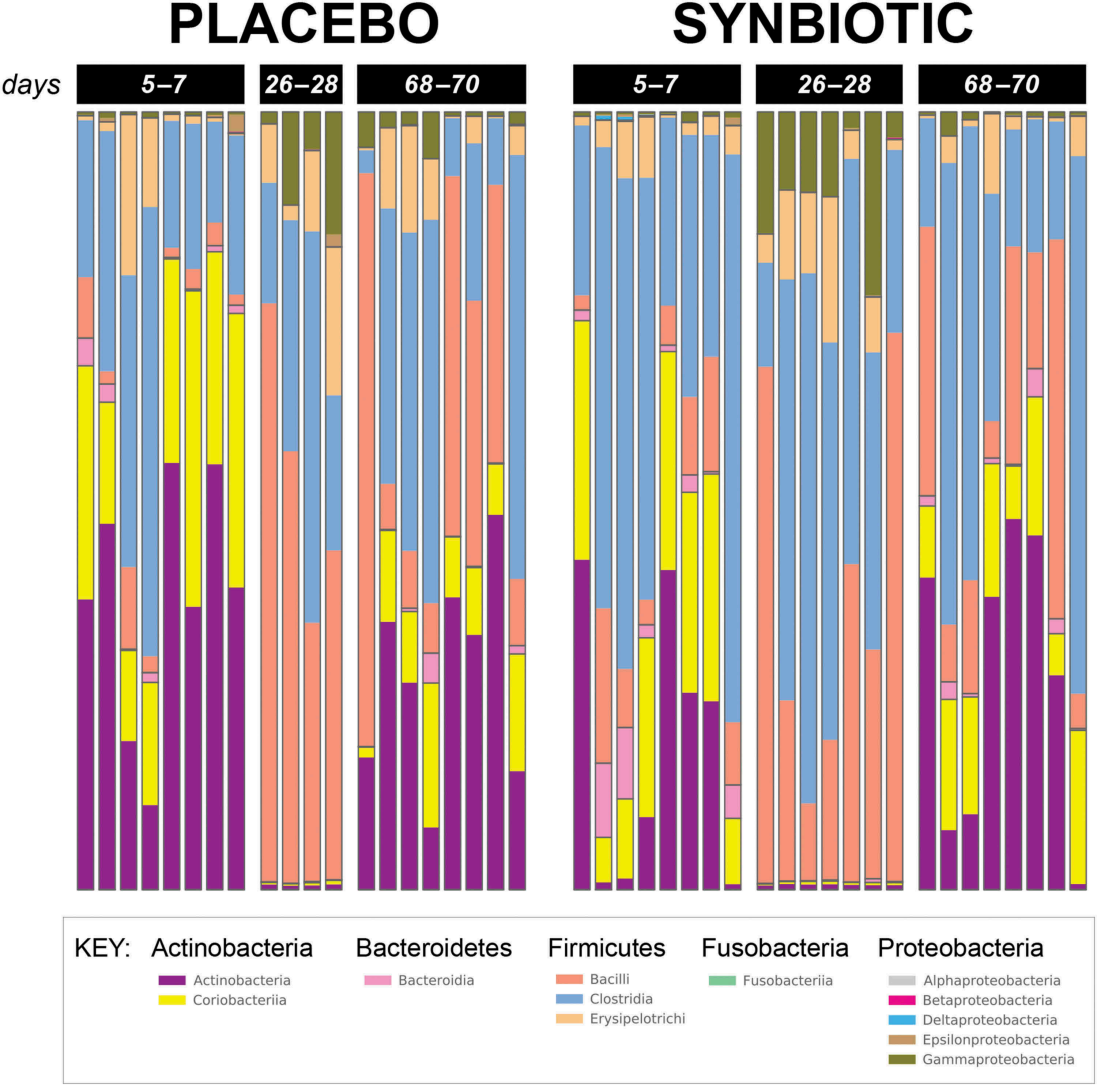
Phylum- and class-level composition of fecal microbiota obtained from feline fecal samples. Samples were collected at baseline (days 5–7), at the conclusion of antibiotic administration (days 26–28), and after a 6-week washout (days 68–70) from 16 healthy cats, 8 per group,^+^ that received 75 mg clindamycin followed 1 h later by either two capsules of a placebo or synbiotic PO once daily for 21 days. ^+^Feces not available from four cats at time point 26–28 because of early termination of treatment due to severe gastrointestinal signs. Legend for all detected classes is shown, grouped by phylum.

Marked differences were identified in the fecal microbiome over time for cats in both treatment groups (Figure 1–2, Table 1, Supplementary Table 1), indicating the majority of changes were due to antibiotic administration. Sex was not significantly associated with changes in any of the analyzed parameters. Age was significantly associated with the dysbiosis index (*F*-value 7.8, *P* = 0.02) and Shannon index (*F*-value 6.6, *P* = 0.02) but not goods coverage (*P* = 0.6) or the Chao1 metric (*P* = 0.6). Age also was associated with relative bacterial abundances at the phylum level over time in the initial statistical model, but these associations did not persist after sex was removed from the model (*P* > 0.05).

**Table 1.** Dysbiosis index and alpha diversity results for cats that received clindamycin and placebo or synbiotic.

	Baseline		Days 26-28		Days 68-70		
	Placebo	Synbiotic	Placebo^+^	Synbiotic	Placebo	Synbiotic	P-value
Dysbiosis index	-3.3^c^	-4.7^c^	1.2^a^	0.6^a^	-2.2^b^	-3.7^b^	<0.01 (time)
	(-5.0 to -2.1)	(-5.5 to -2.2)	(0.8 to 1.6)	(-0.2 to 5.9)	(-4.8 to 0.1)	(-6.2 to -1.7)	
Shannon index	4.5^abc^	5.7^a^	4.0^bc^	4.7^b^	4.8^abc^	4.9^c^	0.03 (group by time),
	(3.9-5.8)	(4.6-6.6)	(3.4-4.3)	(3.5-4.8)	(3.4-5.9)	(4.0-5.8)	<0.01 (time)
Goods coverage	0.9843^b^	0.9832^b^	0.9887^a^	0.9860^a^	0.9843^b^	0.9842^b^	<0.01 (time)
	(0.9781-0.9907)	(0.9784-0.9883)	(0.9866-0.9907)	(0.9831-0.9919)	(0.9799-0.9906)	(0.9809-0.9901)	
Chao1 metric	2,211^a^	2,590^a^	1,644^b^	1,950^b^	2,273^ab^	2,169^ab^	0.02 (time)
	(1,276-3,242)	(1,645-3,324)	(1,307-1,896)	(1,230-2,546)	(1,243-2,933)	(1,206-2,927)	

Median (range) results for feces collected at baseline (days 5–7), at the conclusion of antibiotic administration (days 26–28), and after a 6-week washout (days 68–70) from 16 healthy cats, 8 per group,^+^ that received 75 mg clindamycin followed 1 h later by either two capsules of a placebo or synbiotic PO once daily for 21 days. ^+^Feces not available from four cats at time point 26–28 because of early termination of treatment due to severe gastrointestinal signs. Values that do not share a common superscript letter differed significantly (*P* < 0.05) based on *post hoc* analysis.

Dysbiosis and alpha diversity indices significantly differed over time (Table 1). *Post hoc* tests further revealed that the dysbiosis index was higher on days 26–28 and 68–70 than at baseline, with greater dysbiosis on days 26–28 than 68–70 (*P* < 0.01; *P* < 0.01; *P* < 0.01, respectively). The Shannon index had a significant treatment group by time interaction (*P* = 0.03). *Post hoc* analysis revealed that this was due to significantly lower values at days 26–28 compared to baseline and days 68–70 for each treatment group, with overlap in values among time points for the placebo group but not the synbiotic group. There were no significant associations between treatment group and changes in alpha diversity. Beta diversity was significantly altered on days 26–28 compared to baseline and days 68–70 (*P* < 0.01; R = 0.62) with no significant differences between treatment groups (Figure 1).

Based on sequencing analysis, five phyla were identified (mean baseline relative abundances): *Actinobacteria* (50.54%), *Bacteroidetes* (2.44%), *Firmicutes* (46.12%), *Fusobacteria* (0.04%), and *Proteobacteria* (0.86%). There were marked differences over time for relative abundances of all phyla except *Fusobacteria* for both treatment groups, but differences between treatment groups were not significant (Figure 2, Supplementary Table 1).

Significant treatment group by time differences (*P* = 0.05) in relative bacterial abundance were found for *Microbacteriaceae* and *Turicibacterales*. For *Microbacteriaceae*, *post hoc* analysis revealed that bacterial abundances were significantly higher at days 68–70 for the placebo group than at baseline (baseline, 0.00 [0.00–0.00]; days 26–28 0.00 [0.00–2.86 × 10^–03^], days 68–70, 2.86 × 10^–03^ [0.00–2.86 × 10^–03^]). They were also significantly higher than abundances at days 26–28 or 68–70 for the synbiotic group (baseline, 0.00 [0.00–2.86 × 10^–03^]; days 26–28, 0.00 [0.00–2.86 × 10^–03^]; days 68–70, 0.00 [0.00–0.00]). *Post hoc* analysis revealed that relative abundance of *Turicibacterales* was significantly lower in the placebo group at baseline compared to any other timepoint (baseline, 0.12 [0.08–7.64]; days 26–28, 0.65 [0.46–0.81]; days 68–70, 1.55 [0.15–5.74]), and it also was lower than abundances for the synbiotic group at any timepoint (baseline, 1.08 [0.16–16.08]; days 26–28, 0.43 [0.21–0.73]; days 68–70, 2.87 [0.17–11.34]).

Significant group (*P* = 0.01) and time (*P* < 0.01), but not group by time, differences were identified for *Ruminococcaceae*. Abundances were significantly higher in the synbiotic group (baseline, 3.65 [0.88–6.28]; days 26–28, 0.37 [0.18–2.63]; days 68–70, 0.33 [0.20–0.93]) than the placebo group (baseline, 0.79 [0.23–3.08]; days 26–28, 0.34 [0.13–0.49]; days 68–70, 0.20 [0.15–0.82]) with relative abundances decreasing significantly over time for both treatment groups.

There were 60 operational taxonomic units (OTUs) with significantly different abundances over time alone (Supplementary Table 1). Of these, 20 experienced complete recovery by the conclusion of the washout period, 21 had incomplete return to baseline abundances, 16 had significant decreased abundances during treatment without change thereafter, and three had individual patterns of change.

### Fecal metabolomic profiles

**Figure 3.**
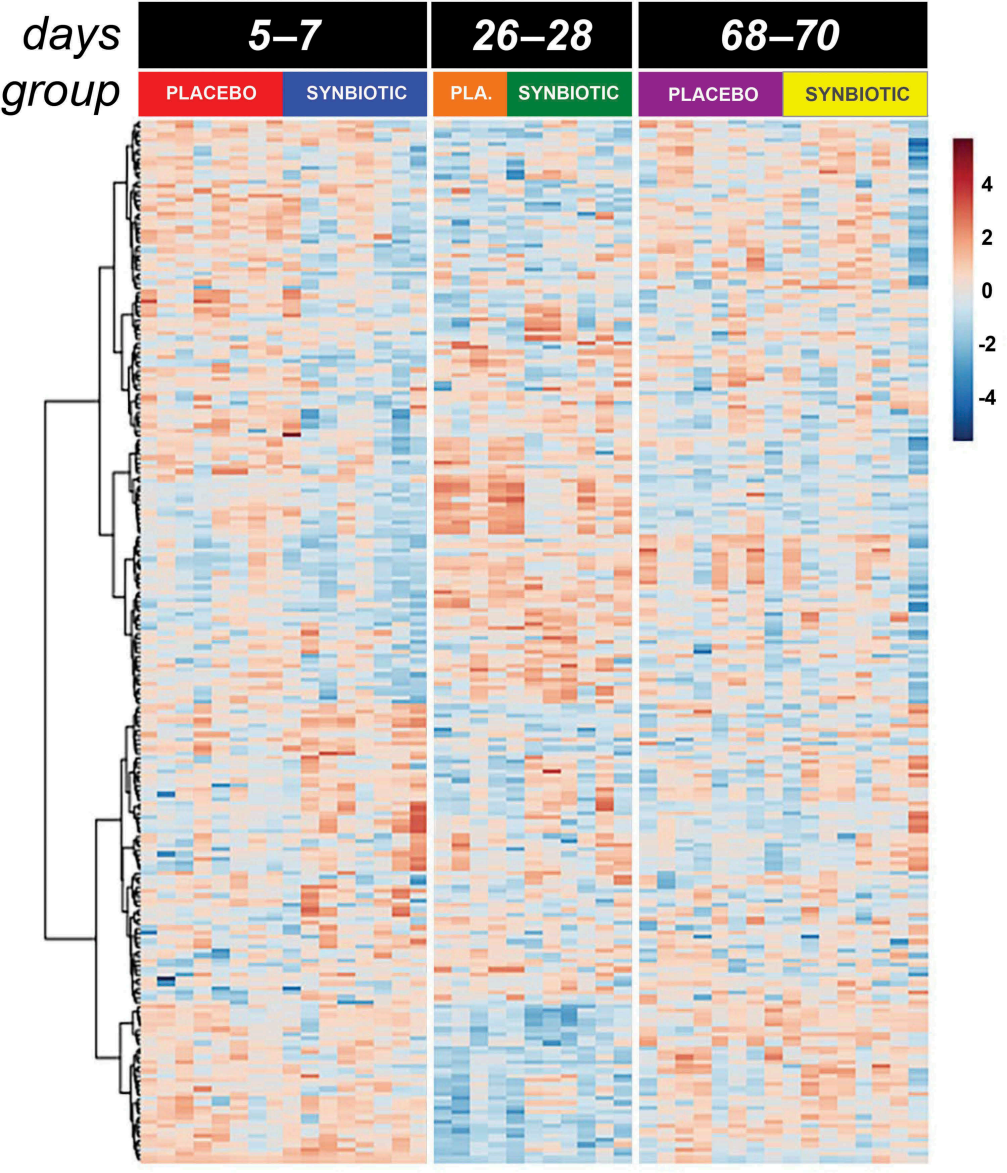
Dual hierarchical dendrogram of metabolites, clustered by pathway, that differed significantly over time in feline fecal samples. Samples were collected at baseline (days 5–7), at the conclusion of antibiotic administration (days 26–28), and after a 6-week washout (days 68–70) from 16 healthy cats, 8 per group,^+^ that received 75 mg clindamycin followed 1 h later by either two capsules of a placebo or synbiotic PO once daily for 21 days. ^+^Feces not available from four cats at time point 26–28 because of early termination of treatment due to severe gastrointestinal signs.

**Figure 4.**
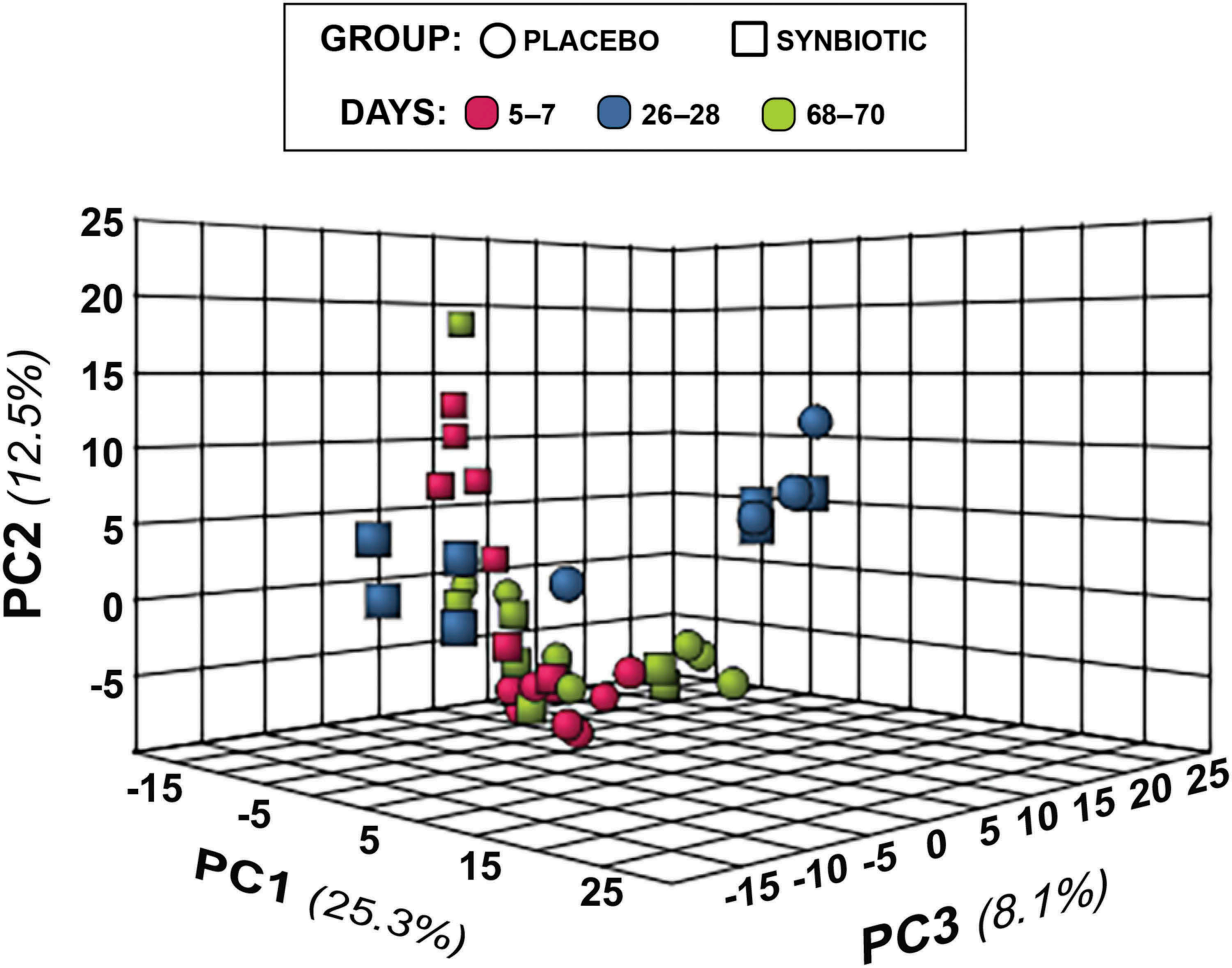
Principal component analysis (PCA) of metabolic pathway analyses from feline fecal samples. Samples were collected at baseline (days 5–7), at the conclusion of antibiotic administration (days 26–28), and after a 6-week washout (days 68–70) from 16 healthy cats, 8 per group,^+^ that received 75 mg clindamycin followed 1 h later by either two capsules of a placebo or synbiotic PO once daily for 21 days. ^+^Feces not available from four cats at time point 26–28 because of early termination of treatment due to severe gastrointestinal signs.

Two hundred fifty-three compounds were identified based on comparison of spectral analysis results to database compounds. Untargeted fecal metabolomic profiles differed markedly over time in both treatment groups (Figure 3–4).

Treatment group by time and treatment group differences were identified for 10 and 5 metabolites (Figure 5a–c, Supplementary Table 2), respectively. Significant group by time interactions were present for 2-deoxytetronic acid, 5,6-dihydrouracil major, dihydrocholesterol, fumaric acid, malic acid, malonic acid, myristic acid, N-acetylaspartic acid, palmitoleic acid, and threitol. In addition to having significant group by time interactions, profiles for six metabolites differed significantly by time (2-deoxytetronic acid, malic acid, malonic acid, myristic acid, N-acetylaspartic acid, and palmitoleic acid) and one (dihydrocholesterol) by treatment group. Significant associations between metabolite profile and treatment group alone were found for 2-hydroxybutanoic acid, myo-inositol, octadecanol, and squalene (though *post hoc* analysis did not clarify the group association for squalene).

**Figure 5.**
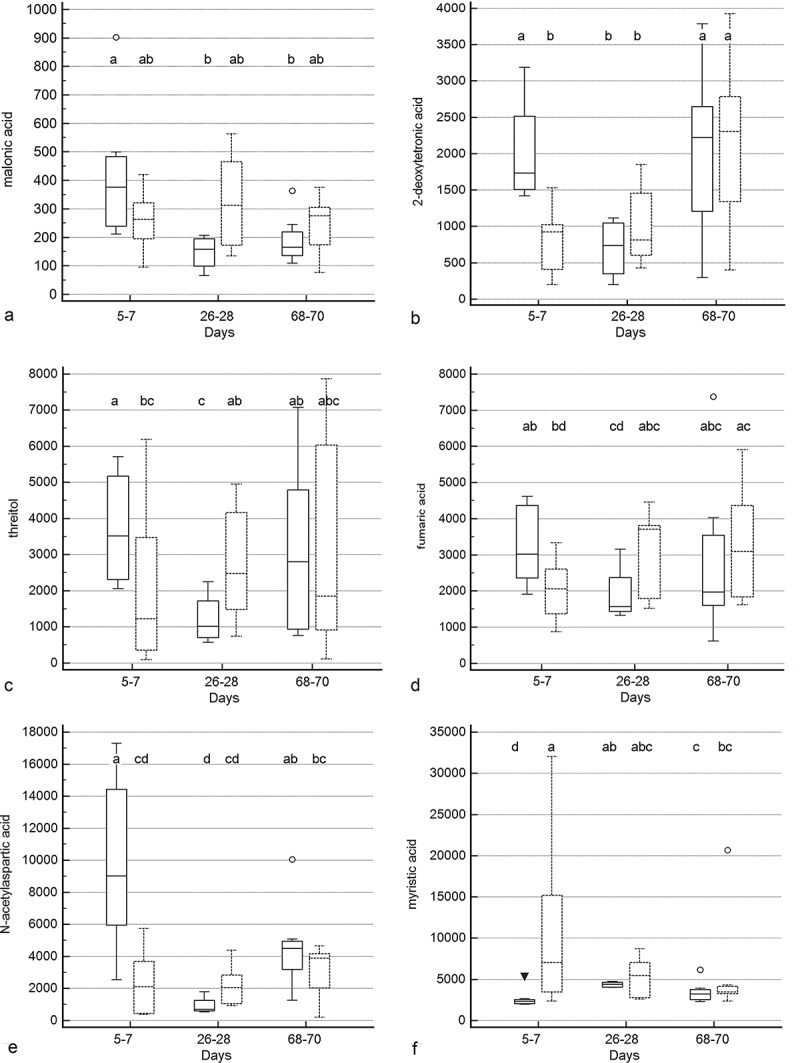
Box and whisker plots of fecal metabolite profiles for selected fecal metabolites that differed significantly (fdr - adjusted *P* < 0.05) between treatment groups and over time. Figure 5a: (A) Malonic acid. (B) 2-deoxytetronic acid. (C) Threitol. (D) Fumaric acid. (E) N-acetylaspartic acid. (F) Myristic acid. Figure 5b: (A) 5,6-dihydrouracil major. (B) Malic acid. (C) Octadecanol. (D) Palmitoleic acid. Figure 5c: (A) Myo-inositol. (B) 2-hydroxybutanoic acid. (C) Squalene. (D) Dihydrocholesterol. Medians, interquartile ranges, and minimum and maximum values are presented for cats in the placebo (boxes with solid borders) and synbiotic (boxes with dashed borders) treatment groups. Open circles and closed triangles denote outlier values. Fecal samples were collected at baseline (days 5–7), at the conclusion of antibiotic administration (days 26–28), and after a 6-week washout (days 68–70) from 16 healthy cats, 8 per group,^+^ that received 150 mg clindamycin with either a placebo or synbiotic PO once daily for 21 days. ^+^Feces from four cats (placebo group) were unavailable at time point 26–28 because of early termination of treatment due to severe gastrointestinal signs. Significance was set as *P* < 0.05, with *P-*values adjusted based on the Benjamini and Hochberg False discovery rate (fdr). Boxes that do not share a letter differed significantly (fdr-adjusted *P* < 0.05) based on *post hoc* analysis. For squalene, *post hoc* analysis did not clarify the group association. Both treatment group and group by time interactions were identified for dihydrocholesterol.

**Figure 5b.**
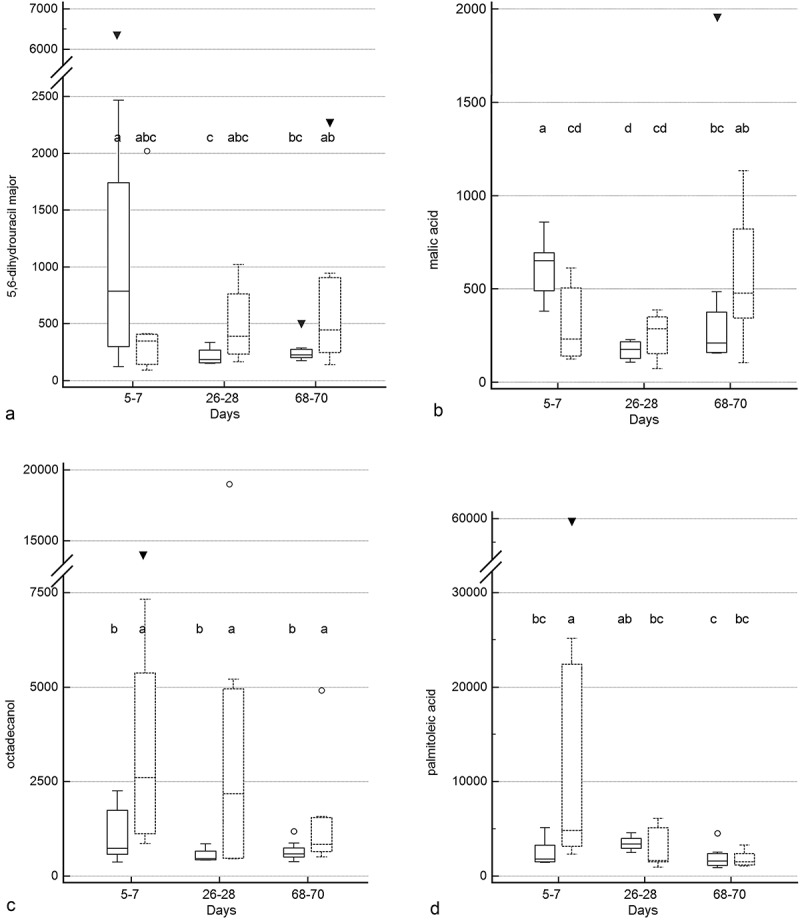
(Continued).

**Figure 5c.**
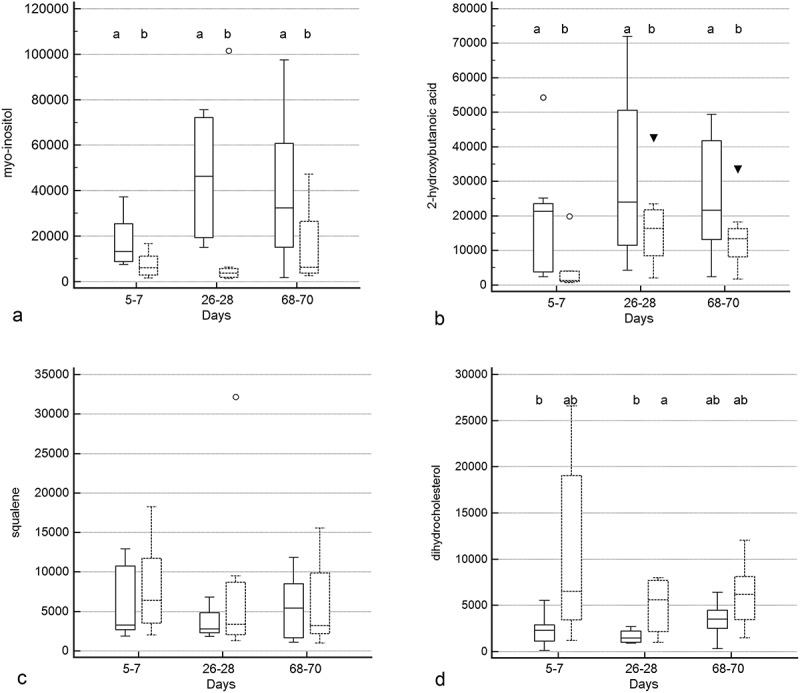
(Continued).

In addition to these associations, significant (fdr-adjusted *P* < 0.05) temporal changes alone were identified for 100 metabolites (Supplementary Table 2), reflecting the impact of antibiotic administration. Of these, profiles for 52 compounds had marked differences between baseline and days 26–28 with full recovery thereafter. Profiles for 12 compounds differed significantly on days 26–28 compared to baseline with partial recovery on days 68–70. An additional 18 compounds had significantly different profiles on days 26–28 compared to baseline without significant change thereafter. Profiles for six metabolites had significantly higher values on days 26–28 compared to baseline but also lower values on days 68–70 compared to the other two time points, with profiles for three metabolites displaying an inverse pattern of overcorrection past baseline. Profiles for nine metabolites were significantly different at days 68–70 compared to baseline and days 26–28, with no difference between profiles for the other two time points.

With regard to metabolites of known biological importance (Table 2), there were significant changes in profiles for SCFA, bile acid, tryptophan, and sphingolipid metabolites, as well as polyamines, antioxidants, and antimicrobials. Significant differences were noted over time for indole-3-acetate profiles, but this association did not retain significance after correction for multiple comparisons (fdr-adjusted *P* = 0.08). Pathway analysis revealed significant temporal alterations in 62 metabolic pathways, 41 of which had impact factors >0 (Table 3). The majority of pathways affected related to SCFA, amino acid, sugar, and nucleotide synthesis and degradation. Tryptophan metabolism pathways were among significantly affected amino acid pathways. Other pathways significantly altered related to energy balance (pyruvate metabolism and citrate cycle), sphingolipids, glycerolipids, and glycerophospholipids.

**Table 2.** Metabolites of known biological import with fecal profiles that significantly differed by group by time interaction, group, and/or time.

	Baseline		Days 26-28		Days 68-70		
	Placebo	Synbiotic	Placebo^+^	Synbiotic	Placebo	Synbiotic	Fdr *P*-value
**Short chain fatty acid metabolites**							
3,4-dihydroxyphenyl-acetic acid	1,615^a^	803^a^	425^b^	828^b^	1,210^a^	2,352^a^	0.04 (time)
	(962-2,680)	(314-3,602)	(223-724)	(383-2,525)	(710-9,369)	(180-20,565)	
3-hydroxyphenyl-acetic acid	529^a^	3,514^a^	78^c^	291^c^	214^b^	252^b^	<0.01 (time)
	(172-4,109)	(289-18,478)	(21-138)	(26-2,221)	(69-12,535)	(152-718)	
2,3-dihydroxy-butanoic acid	697^a^	175^a^	82^b^	50^b^	146^a^	245^a^	0.02 (time)
	(222-1,558)	(52-1,606)	(59-306)	(8-226)	(58-1,832)	(111-901)	
2-hydroxy-butanoic acid	21,383^a^	1,312^b^	24,019^a^	16,403^b^	21,683^a^	13,412^b^	0.05 (group)
	(2,388-54,254)	(702-19,922)	(4,259-71,903)	(2,007-42,590)	(2,317-49,338)	(1,712-33,500)	
3-aminoisobutyric acid	1,567^a^	5,230^a^	2,696^b^	923^b^	5,630^a^	4,558^a^	0.02 (time)
	(832-3,538)	(2,628-6,725)	(89-3,878)	(107-3,548)	(367-7,288)	(929-7,447)	
4-aminobutyric acid	4,798^b^	2,212^b^	40,821^a^	5,644^a^	17,363^a^	12,446^a^	0.01 (time)
	(2,330-111,922)	(1,625-14,553)	(18,349-113,143)	(3,811-66,681)	(6,165-28,013)	(1,236-57,235)	
2,4-diaminobutyric acid	2,291^a^	1,768^a^	312^b^	323^b^	3,413^a^	2,981^a^	<0.01 (time)
	(1,388-5,136)	(366-9,508)	(167-441)	(172-686)	(266-12,630)	(635-6,901)	
4-hydroxybutyric acid	2,269^a^	971^a^	1,751^a^	1,876^a^	1,041^b^	905^b^	0.01 (time)
	(644-8,014)	(452-3,697)	(1,199-3,123)	(1,198-2,240)	(343-1,796)	(273-1,203)	
3-phenyllactic-acid	5,318^b^	1,366^b^	4,709^b^	4,363^b^	26,433^a^	16,257^a^	<0.01 (time)
	(523-20,918)	(275-12,731)	(4,529-23,160)	(442-12,880)	(2,799-50,765)	(185-44,885)	
Lactic acid	29,969^b^	6,457^b^	1,046,986^a^	288,190^a^	473,391^a^	188,366^a^	0.01 (time)
	(4,279-1,912,722)	(3,129-224,210)	(897,705-1,199,654)	(19,338-1,514,659)	(4,123-1,692,997)	(2,810-1,067,482)	
P-hydroxylphenyl-lactic acid	2,775^c^	712^c^	3,183^b^	6,196^b^	15,378^a^	10,557^a^	<0.01 (time)
	(1,076-8,670)	(332-3,808)	(1,372-5,234)	(2,315-16,911)	(3,039-33,947)	(469-42,710)	
Propane-1-3-diol	274^b^	354^b^	4,287^a^	3,278^a^	1,696^a^	1,375^a^	<0.01 (time)
	(194-7,900)	(161-653)	(2,020-4,881)	(1,375-6,740)	(495-16,144)	(367-11,753)	
3,3-hydroxyphenyl propionic acid	118,316^a^	139,062^a^	132^c^	190^c^	1,238^b^	948^b^	<0.01 (time)
	(55,264-160,927)	(55,351-212,599)	(92-377)	(44-1,634)	(188-190,172)	(170-181,388)	
3-(4-hydroxyphenyl)-propionic acid	41,265^a^	71,768^a^	4,156^c^	4,981^c^	18,509^b^	17,346^b^	<0.01 (time)
	(24,170-110,451)	(34,626-143,552)	(1,907-10,735)	(1,442-18,082)	(5,641-48,155)	(4,827-33,522)	
Succinic acid	48,717^b^	2,100^b^	195,371^ab^	40,189^ab^	218,304^a^	299,338^a^	0.03 (time)
	(1,935-390,402)	(747-271,563)	(21,751-795,120)	(8,920-1,147,221)	(1,166-674,751)	(2,113-619,092)	
**Bile acids**							
Dihydrocholesterol	2,279^b^	6,518^ab^	1,446^b^	5,581^a^	3,528^ab^	6,203^ab^	<0.01 (group by time), <0.01 (group)
	(100-5,528)	(1,182-26,585)	(863-2,691)	(964-7,994)	(281-6,382)	(1,448-12,017)	
Deoxycholic acid	78,243^a^	38,492^a^	299^c^	1,358^c^	8,383^b^	4,594^b^	<0.01 (time)
	(838-288,348)	(8,421-922,997)	(81-1,329)	(202-16,475)	(84-298,628)	(35-976,489)	
**Tryptophan metabolites**							
Indole-3-lactate	110,344^a^	134,566^a^	26,768^b^	75,418^b^	117,419^a^	162,374^a^	0.01 (time)
	(72,690-224,874)	(33,221-453,636)	(13,271-102,119)	(44,683-184,909)	(65,124-188,639)	(1,430-338,952)	
**Sphingolipid metabolites**							
Cellobiose	21,065^a^	4,832^a^	49,231^a^	5,255^a^	2,774^b^	3,282^b^	0.01 (time)
	(2,701-39,188)	(318-37,705)	(8,116-247,769)	(3,566-11,868)	(823-6,438)	(271-8,657)	
Isopentadecanoic acid	41,428^a^	92,495^a^	30,204^b^	24,276^b^	19,356^b^	14,396^b^	<0.01 (time)
	(25,355-126,295)	(44,950-173,882)	(26,776-49,252)	(10,430-34,491)	(4,918-256,801)	(4,901-116,494)	
Pentadecanoic acid	25,635^a^	65,751^a^	15,268^b^	10,376^b^	9,767^b^	12,034^b^	<0.01 (time)
	(17,767-96,730)	(19,963-118,284)	(5,939-17,510)	(9,721-13,001)	(5,710-13,775)	(6,110-46,327)	
**Polyamines**							
Putrescine	592,861^a^	400,585^a^	165,171^b^	82,950^b^	192,405^b^	221,571^b^	0.05 (time)
	(188,643-1,084,643)	(7,787-2,741,972)	(66,399-416,065)	(11,840-672,670)	(16,170-725,961)	(49,993-742,246)	
Squalene†	3,289 (1,872-12,906)	6,399 (2,022-18,225)	2,808 (1,806-6,805)	3,397 (1,248-32,188)	5,418 (1,087-11,839)	3,230 (956-15,542)	<0.01 (group)
**Antioxidants / antimicrobials**							
3,4-dihydroxyhydrocinnamic acid	27,522^b^	14,896^b^	33,604^ab^	38,149^ab^	40,648^a^	148,715^a^	0.04 (time)
	(764-39,566)	(1,307-39,007)	(11,036-44,143)	(17,192-78,347)	(1,836-363,292)	(1,501-640,134)	
3,4-dihydroxybenzoic acid	10,816^a^	4,262^a^	2,831^b^	3,029^b^	7,107^a^	19,317^a^	0.05 (time)
	(3,732-21,603)	(2,483-20,760)	(2,069-4,290)	(2,092-3,525)	(2,617-33,854)	(1,085-48,873)	
4-hydroxybenzoate	22,974^a^	11,663^a^	4,288^b^	4,153^b^	16,556^a^	19,013^a^	0.02 (time)
	(15,351-46,996)	(1,061-24,039)	(1,355-11,641)	(1,726-14,652)	(5,239-44,331)	(1,301-41,178)	

Median (range) peak height of metabolites in feces collected at baseline (days 5–7), at the conclusion of antibiotic administration (days 26–28), and after a 6-week washout (days 68–70) from 16 healthy cats, 8 per group,^+^ that received 75 mg clindamycin followed 1 h later by either two capsules of a placebo or synbiotic PO once daily for 21 days. ^+^Feces not available from four cats at time point 26–28 because of early termination of treatment due to severe gastrointestinal signs. Fdr *P*-value = Benjamini and Hochberg False discovery rate (fdr) adjusted *P*-value. Profiles that do not share a common superscript letter differed significantly based on *post hoc* analysis. ^†^*Post hoc* analysis did not clarify source of significant differences between groups.

**Table 3.** Metabolic pathways that differed significantly over time.

		Total Cmpds	Hits	Fdr *P*-value	Pathway impact value
1*	Alanine, aspartate and glutamate metabolism	24	10	9.55E-04	0.59
2*	Glycine, serine and threonine metabolism	48	9	5.91E-08	0.54
3°	Galactose metabolism	41	14	1.93E-05	0.52
4*	Arginine and proline metabolism	77	16	1.70E-04	0.48
5*	Taurine and hypotaurine metabolism	20	4	4.27E-04	0.38
6°	Starch and sucrose metabolism	50	8	9.52E-04	0.36
7*	Beta-Alanine metabolism	28	6	6.84E-04	0.34
8	Pyruvate metabolism	32	2	5.02E-03	0.32
9*	Phenylalanine metabolism	45	13	6.49E-07	0.27
10†	Pantothenate and CoA biosynthesis	27	6	8.59E-05	0.25
11*	Cysteine and methionine metabolism	56	7	6.49E-07	0.23
12*	Lysine degradation	47	4	1.86E-04	0.23
13‡	Pyrimidine metabolism	60	9	4.01E-05	0.22
14	Glycerolipid metabolism	32	5	7.93E-06	0.22
15	Histidine metabolism	44	4	1.39E-02	0.21
16	Citrate cycle (TCA cycle)	20	4	1.75E-02	0.21
17°‡	Amino sugar and nucleotide sugar metabolism	88	9	1.17E-02	0.21
18*	Tyrosine metabolism	76	10	4.01E-05	0.20
19	Aminoacyl-tRNA biosynthesis	75	18	9.70E-08	0.17
20†	Butanoate metabolism	40	8	6.19E-03	0.17
21*	Tryptophan metabolism	79	2	2.88E-02	0.16
22‡	Purine metabolism	92	13	5.40E-09	0.12
23*	Phenylalanine, tyrosine and tryptophan biosynthesis	27	5	1.49E-05	0.11
24*	Lysine biosynthesis	32	3	1.11E-04	0.10
25	Glycolysis or Gluconeogenesis	31	4	3.49E-03	0.10
26	Sphingolipid metabolism	25	3	5.32E-03	0.09
27°	Pentose and glucuronate interconversions	53	7	3.81E-02	0.09
28	Glycerophospholipid metabolism	39	2	6.52E-04	0.09
29†	Propanoate metabolism	35	6	2.81E-04	0.09
30*	Valine, leucine and isoleucine biosynthesis	27	6	1.15E-06	0.09
31	Sulfur metabolism	18	3	9.70E-08	0.07
32°	Fructose and mannose metabolism	48	3	2.55E-02	0.07
33	Nitrogen metabolism	39	10	1.47E-04	0.07
34*	Valine, leucine and isoleucine degradation	40	4	7.03E-06	0.06
35	Ubiquinone and other terpenoid-quinone biosynthesis	36	4	4.27E-04	0.05
36	Glyoxylate and dicarboxylate metabolism	50	5	5.68E-03	0.04
37	Caffeine metabolism	21	1	2.37E-04	0.03
38	Fatty acid metabolism	50	2	5.17E-03	0.03
39	Glutathione metabolism	38	5	4.01E-05	0.03
40°	Pentose phosphate pathway	32	4	3.83E-03	0.02
41	Methane metabolism	34	2	8.82E-06	0.02

Pathways that differed significantly over time by group based on fecal metabolite profiles in feces collected at baseline (days 5–7), at the conclusion of antibiotic administration (days 26–28), and after a 6-week washout (days 68–70) from 16 healthy cats, 8 per group,^+^ that received 75 mg clindamycin followed 1 h later by either two capsules of a placebo or synbiotic PO once daily for 21 days. ^+^Feces not available from four cats at time point 26–28 because of early termination of treatment due to severe gastrointestinal signs. Total Cmpds = total compounds in pathway; fdr *P*-value = Benjamini and Hochberg False discovery rate (fdr) adjusted *P*-value; * = amino acid metabolism,  = sugar metabolism, † = short-chain fatty acid metabolism, ‡ = nucleotide metabolism.

## Discussion

Consistent with previous work,^1^ administration of clindamycin induced profound but reversible alterations in the fecal microbial alpha and beta diversity of cats, as well as long-lasting derangements in the fecal microbiome and metabolome. Significant age-related associations were identified for the dysbiosis index, Shannon index, and relative bacterial abundances at the phylum level, although the latter association did not retain significance after removal of sex from the model. Sex was not associated with any of the analyzed factors, in spite of the strong association between female sex and vomiting in the previously published study of AAGS.^2^ In spite of the loss of power for one time point in this study, significant differences were identified between treatment groups for the Shannon index, relative bacterial abundance of *Ruminococcaceae*, and profiles for a number of fecal metabolites (2-hydroxybutanoic acid, dihydrocholesterol, myo-inositol, octadecanol, squalene, 2-deoxytetronic acid, 5,6-dihydrouracil major, dihydrocholesterol, fumaric acid, malic acid, malonic acid, myristic acid, N-acetylaspartic acid, palmitoleic acid, and threitol). Some were for metabolites or pathways of known biological significance, such as SCFA, bile acids, and polyamines. These differences could have contributed to previously reported period effects for food intake, vomiting, and successful completion of treatment.^2^ Further evaluation would be necessary to determine which, if any, of these factors contributed to differences in individual clinical signs between treatment groups.

Marked reductions in relative abundances of *Actinobacteria* (*Adlercreutzia, Bifidobacterium, Collinsella, Slackia*), *Bacteroidia* (*Bacteroides, Prevotella*), *Ruminococcaceae* (*Faecalibacterium, Ruminococcus*), *Veillonellaceae* (*Megamonas, Megasphaera, Phascolarctobacterium*) and *Erysipelotrichaceae* ([*Eubacterium*]) were noted during antibiotic administration, as were increases in *Clostridiaceae* (*Clostridium*) and *Proteobacteria* (*Enterobacteriaceae*). Changes during antibiotic administration mostly were consistent with previous reports,^1,3^ although patterns at the conclusion of washout differed from those previously reported for a number of OTUs. Significant treatment group by time interactions were identified in this study, but not a prior study^1^ that used the same antibiotic and synbiotic, for relative abundances of *Microbacteriaceae* and *Turicibacterales*. These associations, however, were weak (fdr-adjusted *P* = 0.05). Based on the review of the relative abundances, the association for *Microbacteriaceae* likely reflects type 1 error given marginal statistical significance and extremely low relative abundances of the OTU in both treatment groups at all timepoints. Other significant changes in relative abundances noted in this study, but not the former one, were time-related changes in abundances of *Bacilli, Bacillales, Lactobacillales, Episilonproteobacteria, Campylobacterales, Campylobacteraceae, Gammaproteobacteria, Enterobacteriales*, and *Enterobacteriaceae*. Conversely, the previous study found significant temporal changes in relative abundances of *Coprococcus, Dorea, Roseburia*, and *Oscillospira*, none of which occurred in this study. A separate study^3^ identified significant changes in *Dialister* and *Enterococcus* abundances in the feces of cats during treatment with amoxicillin-clavulonate with or without *Enterococcus faecium* SF68, neither of which was noted in this study.

Discordance among the three studies in changes for some OTUs could reflect the use of differing antibiotics and dosages, probiotics/synbiotics and dosages, and timing of probiotic/synbiotic administration, as well as underpowering in the current study due to the limited number of samples for the placebo group for days 26–28. Other differences might be explained by the marked difference in the terminal sampling time points among the studies (7, 40–42, and 603–605 days after antibiotic discontinuation). For example, *Actinobacteria, Actinomycetales, Actinomycetaceae, Coriobacteriia, Coriobacteriales, Coriobacteriaceae, Slackia, Bacteroidetes, Bacteroidia*, and *Bacteroidales* spp had partial return to baseline abundances after antibiotic discontinuation in this study, but abundances completely returned to baseline values by days 603–605 of recovery in one study.^1^ Additionally, complete or partial return to baseline abundances by days 603–605 of recovery was noted for *Prevotellaceae, Prevotella, Clostridiales, Clostridiaceae, Peptococcaceae, Peptococcus, Ruminococcaceae, Faecalibacterium, Veillonellaceae, Megaphaera*, and *Phascolarctobacterium* spp in the previous work, but no change was noted in relative abundances between treatment and days 40–42 of recovery in this study. Conversely, partial or complete return to baseline abundances was noted in this study for a number of OTUs that had overcorrection past baseline abundances at days 603–605 of recovery in the previous study. These included *Adlercreutzia, Bacteroidaceae, Bacteroides, Porphyromonadaceae, Parabacteroides, SMB53, Ruminococcus, Campylobacteraceae, Gammaproteobacteria, Enterobacteriales*, and *Enterobacteriaceae* spp. Ongoing changes in abundance for many OTUs could reflect persistent, potentially irrevocable, dysbiosis secondary to antibiotic exposure. These changes are particularly intriguing given associations between personal or maternal antibiotic use and risk of future AAGS, inflammatory bowel disease, diabetes mellitus, and obesity in people.^7–12^ Continued shift in bacterial populations long after discontinuation of antibiotic therapy also partially reflect aging changes in the microbiome,^13,14^ although no association was found between age and baseline microbiome characteristics in this or another study.^1^

In spite of differences in temporal patterns of change for individual OTUs between this and one previous study,^1^ metabolomic effects were remarkably similar. Many of the same derangements also were found in healthy dogs administered metronidazole for 14 days.^15^ Metabolite profiles and pathways significantly altered by antibiotic administration in this study included SCFA, bile acid, tryptophan, sphingolipid, polyamine, antioxidant, and antimicrobial metabolite profiles. These metabolites and metabolic pathways have important roles in immunomodulation; maintenance of intestinal motility, barrier function, and eubiosis; decreasing production of adherence and virulence factors, motility, biofilm formation, and adherence of pathogenic *E. coli*; and increasing intestinal mucin production, bacterial clearance, and resistance to oxidative stress.^16–23^ Derangements in SCFA, bile acid, tryptophan, and sphingolipid and phospholipid profiles also have been associated with AAGS in people,^24,25^ suggesting a causal link between these abnormalities and AAGS in cats.

The combined alterations in the microbiome and polyamine synthesis in this study, as well as prior work, are particularly intriguing. Polyamines increase intestinal epithelial restitution after injury,^26^ decrease epithelial mucosal sensitivity to apoptosis,^27^ and decrease inflammatory cytokine production.^28^ The majority of fecal polyamines appear to be synthesized by gut microbiota, including *Bacteroides* spp, *Fusobacterium* spp, and members of the *Clostridium* subcluster XIVa, including *Clostridum, Roseburia, Eubacterium*, and *Ruminococcus* spp.^29–31^ Bacterial polyamine synthesis can be enhanced both by administration of prebiotics^29^ and probiotics.^32–34^ Increases in fecal polyamine concentrations after consumption of probiotic-containing yogurt correlated with marked reductions in haptoglobin and fecal mutagens in two studies in elderly people.^33,34^ In one study of dogs with inflammatory bowel disease, mucosal putrescine and spermine expression were significantly reduced compared to normal dogs.^35^ Excitingly, administration of probiotics was associated with simultaneous normalization of clinical signs, histology, and putrescine expression in that study. Unfortunately, neither fecal polyamine concentrations nor prior antibiotic exposure was determined.

In this study, marked reductions in relative abundance of *Bacteroides* spp and members of the *Clostridium* subcluster XIVa occurred, and abundances for *Ruminococcaceae* spp significantly differed between treatment groups. Profiles for squalene and putrescine differed significantly over time, with concurrent significant differences between treatment groups for squalene. Similar changes were found in the previously mentioned studies of the microbiome^1,3^ and metabolome^1^ of cats administered antibiotics with or without pro- or synbiotics. In the latter study, putrescine profiles were persistently reduced in cats administered clindamycin with a placebo while similar suppression was not identified in cats administered a synbiotic. Squalene previously has been found to inhibit ornithine decarboxylase,^36^ which is responsible for conversion of ornithine to putrescine, the first of several polyamines. Because squalene is an intermediate in the biosynthesis of cholesterol,^36^ alterations in its profiles also must be considered in concert with noted changes for dihydrocholesterol profiles. As such, concomitant quantification of bacterial changes and polyamine concentrations in the mucosa and feces of animals administered antibiotics with and without synbiotics will be necessary to determine whether ameliorative effects of synbiotics on AAGS are mediated through maintenance of polyamine concentrations.

There are a number of limitations to this study. A crossover design was employed in the previously reported study of AAGS.^2^ However, inadequate washout was identified in that study, based on the presence of period effects. As a result, samples from the last time point were not included in our fecal microbiome and metabolome assessments, resulting in modification of the statistical analysis from a mixed repeated measures crossover design to a standard split-plot repeated measures using the first treatment period. The end result was a limited duration of follow-up after discontinuation of antibiotics and synbiotics, complicating comparison with previously published data.^1^ Comparison between studies also is complicated by differences in the antibiotic and synbiotic dosages used, as well as the timing of synbiotic administration relative to antibiotic administration. The absence of fecal data on days 26–28 for four cats in the placebo group because of early discontinuation of treatment due to severe AAGS decreased the power of the study. In spite of this unanticipated loss of power, significant differences in the fecal microbiome and metabolome were confirmed among time points and between treatment groups. The majority of cats in this study were of advanced middle-age and had historical antibiotic exposure, which could have sensitized them to development of AAGS.^7,37^ Differing results might be found in younger cats and cats without prior antibiotic exposure. Additionally, the use of healthy research cats with a uniform environment, husbandry, and minimal differences in diet might limit extrapolation of results to privately owned cats. Finally, 16S rRNA sequencing was used to characterize the fecal microbiome, which allows determination of the relative but not actual abundance of OTUs.^38^ Broad-spectrum antibiotics, such as clindamycin, markedly decrease gastrointestinal bacterial abundance overall. However, because some microorganisms have inherent antibiotic resistance, their absolute abundance could remain steady in spite of differences in relative bacterial abundance.^38^ Additionally, use of 16 rRNA alone for sequencing prevents characterization of archaea, fungi, protists, and viruses,^38,39^ which also contribute to the microbiome and can effect host- and microbial-changes .

In conclusion, administration of clindamycin at a dosage of 18.0 mg/kg (range 12.1–22.7 mg/kg) PO once daily to healthy cats receiving either a placebo or synbiotic resulted in dysbiosis with marked alterations in fecal alpha and beta diversity. It also was associated with significant changes in relative bacterial abundances of many OTUs, including *Bacteroides* spp and members of the *Clostridium* subcluster XIVa, with significant differences in abundances for *Ruminococcaceae* spp between treatment groups. Changes in abundances for many OTUs persisted at least 6 weeks after antibiotic discontinuation. Metabolite profiles also were significantly altered by antibiotic administration, including profiles related to SCFA, bile acid, tryptophan, sphingolipid, polyamine, antioxidant, and antimicrobial metabolic pathways. Significant treatment group and treatment group by time differences were identified between cats in the placebo *vs*. synbiotic group for *Ruminococcaceae* and some SCFA, bile acid, and polyamine pathway metabolites. Of particular note were changes related to polyamine production. Further investigation is warranted to elucidate their role in prevention of AAGS in cats, people, and other animals treated with synbiotics.

## Materials and methods

### Study design

**Figure 6.**
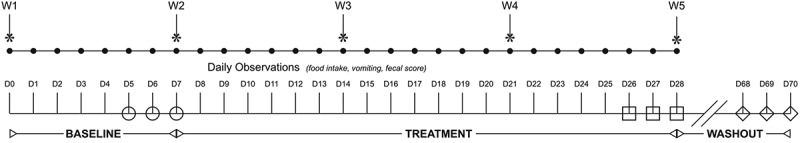
Study design, duration, observations, and sampling flowchart. The study spanned 670 days (D1–70) and was broken into three study periods: baseline (D1–D7), treatment (D8–D28), and washout (D29–70). Cats were randomized to receive 75 mg clindamycin PO followed 1 h later by either a placebo or synbiotic once daily during treatment. Food intake, vomiting, and fecal score were recorded daily (•) and weight (W) weekly (*) by an individual blinded to treatment group. From the center portion of each first morning naturally voided fecal sample for each cat, 2 g were collected once daily on the last 3 days of each study period: baseline (open circles), treatment (open squares), and recovery (open diamonds). Each sample was subdivided into two aliquots, with each aliquot placed into a 2 mL cryovial and immediately frozen at –80ºC pending completion of data collection.

Fecal samples collected during the first phase of a previously reported randomized, double-blinded, placebo-controlled, 2-way, 2-period, cross-over study with a 6-week washout period^2^ were used for this study (Figure 6). The study protocol was approved by the Institutional Animal Care and Use Committee of the University of Tennessee, Knoxville (protocol number 2375) and performed in compliance with “The Guide for the Care and Use of Laboratory Animals” in laboratory animal facilities that are AAALAC certified and exceed NIH standards of care.

Briefly, 16 healthy research cats were randomized using a random number sequence to two treatment groups after stratification by prior antibiotic exposure (12 cats received antibiotics >2.5 years prior to the start of the study and two cats within the previous 6 months). Each study period consisted of a 1-week baseline and a 3-week treatment period. Cats received 75 mg clindamycin (median dose 18.0 mg/kg/d, range 12.1–22.7 mg/kg/d) PO once daily after eating their daily ration of commercial food for 3 weeks, followed 1 h later by either two capsules of a synbiotic (Proviable-DC®, Lot #45222, Nutramax Laboratories Veterinary Sciences, Inc., Lancaster, SC USA) or a placebo. The synbiotic contains 5 billion *cfu* of a proprietary mixture of *Bifidobacterium bifidum, Enterococcus faecium* and *thermophilus*, and *Lactobacillus acidophilus, bulgaricus, casei*, and *lantarum* per capsule, as well a proprietary blend of fructooligosaccharide and arabinogalactan. Opaque empty gelatin capsules (#3 White Capsules, Catalog #684623, Lot #1407280052, LetCo Medical, Decatur, AL USA) of similar size and color as the synbiotic capsules were used as placebos. Cats were maintained on their individual maintenance diet plans throughout the study.

### Fecal samples

First morning naturally voided fecal samples were collected daily for 3 days for each cat at each time point (days 5–7, 26–28, 68–70, and 89–91) to minimize the effects of daily variation and differential distribution of bacterial groups and metabolites within individual fecal samples on results. A 2-g sample was taken from the center portion of each fecal sample and subdivided into two aliquots, placed into individual 2 mL cryovials, and immediately frozen at –80ºC pending completion of data collection. Because significant period effects were identified in AAGS in the prior study,^2^ consistent with an inadequate clinical washout period, samples taken after completion of the second treatment phase (days 89–91) were excluded from this study. Samples for each cat from each time point (days 5–7, 26–28, and 68–70) were combined directly prior to sample analysis to generate pooled samples for microbiome and metabolomic analysis.

### Microbiome analysis

Fecal microbiome analysis was performed as per a previous study.^1^ Briefly, genomic DNA was extracted from 100 mg of feces using a commercially available kit according to manufacturer’s protocol (PowerSoil®, Mo Bio, Catalog #12888–100). The V4 variable region (primers 515F/806R) of the 16S rRNA gene was amplified and sequenced using a MiSeq (Illumina) at a sequencing facility (MR DNA (Molecular Research LP), Shallowater, TX, USA).^40^ Processing and analysis of sequences was performed using Quantitative Insights Into Microbial Ecology (QIIME, v. 1.8. Available at: http://www.qiime.org). Raw sequence data were de-multiplexed and low quality reads filtered using default parameters. Chimeric sequences were detected using USEARCH against the 97% clustered representative sequences from the Greengenes database (v. 13.8) and removed prior to further analysis. Remaining sequences were assigned to OTUs using the uclust consensus taxonomy assigner, default QIIME parameters, and the Greengenes database (v. 13.8), then rarefied to 35,000 sequences per sample. The sequences were deposited in SRA (https://www.ncbi.nlm.nih.gov/sra) under accession number SRP08225.

Quantitative PCR was performed for total bacterial DNA, *Faecalibacterium* spp., *Turicibacter* spp., *Streptococcus* spp., *Escherichia coli, Blautia* spp., *Fusobacterium* spp., and *Clostridium hiranonis* as previously described.^1,41^ For this, 5 μl of a DNA-binding dye,^h^ 0.4 μl each of a forward and reverse primer (final concentration: 400 nM), and 2.6 μl of PCR water were combined with 2 μl of normalized DNA (final concentration: 5 ng/μl) for a total reaction volume of 10 μl. Data were expressed as log amount of DNA (fg) for each particular bacterial group per 10 ng of isolated total DNA. Oligonucleotide primers and probes, as well as respective annealing temperatures, were as previously described.^1^

### Fecal metabolomics

A metabolomics facility (West Coast Metabolomics Core, University of California, Davis, CA, USA) analyzed 10 mg of lyophilized feces from each sample using gas chromatography time-of-flight mass spectrometry (GC-TOF-MS) and standardized protocols.^42,43^ ChromaTOF v. 2.32 was used to process raw data. BinBase algorithm was applied to match spectra to database compounds or characterize spectra as an unknown compound, and quantification was reported by peak height of an ion at the specific retention index characteristic of the compound across all samples. The metabolomics data were deposited in the Metabolomics Workbench repository (http://metabolomicsworkbench.org) under project PR000669, study ST000981.

### Statistical analyses

Descriptive statistics were generated for each response measure. Samples were analyzed for normality using the Shapiro–Wilk test and for the presence of outliers using box-and-whisker plots. A dysbiosis index was calculated.^41^ QIIME scripts were used to create alpha rarefaction plots, as well as calculate measures of alpha diversity (Chao1, Shannon, Goods Coverage, and Observed Species). Beta diversity was determined using unweighted Unifrac distance metrics; principal coordinates analysis (PCoA) plots and rarefaction curves were plotted using QIIME software. The ANOSIM function in PRIMER 6 (PRIMER 6, PRIMER-E Ltd) was used to compare beta diversity across time and groups of cats.^44^ Global changes in untargeted metabolomic profiles were determined using principal component analysis (PCA) plots generated using an online metabolomics software analysis suite (MetaboAnalyst 3.0. Available at: http://www.metaboanalyst.ca).^45,46^ Pathway analysis was performed in the same software suite using the *Homo sapiens* pathway library, interquantile range data filtering, log transformation, and Pareto scaling.

The dysbiosis index, Shannon indexes, goods coverage, Chao 1 metric, relative abundances of bacteria groups, and fecal metabolite profiles were compared between treatment groups using split-plot repeated measures ANOVAs that included fixed effects of treatment group (placebo or synbiotic), time period, and treatment group by time period interaction. Age and sex were included as covariates in the initial analyses. Sex was not retained in any of the final models due to lack of significant effects. Age was significantly associated with the dysbiosis index, Shannon index, and relative bacterial abundances at the phylum level over time. However, once sex was removed from the models, age only retained significance for the dysbiosis index and Shannon indices (see Results). Because covariate results were inconsistent, they were removed from the final models for analyses of bacterial relative abundances to help ensure Type I errors were not committed. The repeated measure of time period was accounted for in a repeated statement and random effects for cat nested within group were included. A compound symmetry variance/covariance structure was incorporated into each model to account for the potential inclusion of constant covariates over time. The Shapiro–Wilk test of normality of the residuals was evaluated for each marker to confirm the assumption of normally distributed residuals had been met. Model assumptions regarding equality of variances were verified with the Levene’s test for equality of variances. *Post hoc* differences in least squares means were determined for markers with significant main effect or interaction terms. Due to marked heterogenous variability in microbiome measures for cats on days 26–28 (see Results), a rank transformation had to be employed to allow convergence of the model for analysis of the proportions of bacteria groups and ensure the statistical assumptions regarding normally distributed residuals and equality of variances were met. Only bacteria taxa that were present in at least 50% of cats in ≥1 group at ≥1 time point were included in statistical analyses.

*P* < 0.05 was considered statistically significant. *P*-values were corrected for multiple comparisons on each phylogenetic level and for untargeted metabolomics using the Benjamini and Hochberg’s False Discovery Rate (fdr). Publicly accessible software packages (MetaboAnalyst 3.0. Available at: http://www.metaboanalyst.ca; MedCalc 15.8 MedCalc Software; SAS 9.4 release TS1M3, SAS Institute Inc.; and SAS Software version 9.4 release TS1M3) were used for all analyses.
