# Supplementary material for: Effects of a synbiotic on the fecal microbiome and metabolomic profiles of healthy research cats administered clindamycin: a randomized, controlled trial
Source: Gut Microbes. 2019 Feb 1;10(4):521–39. doi: 10.1080/19490976.2018.1560754 (PMC6748608; doi:10.1080/19490976.2018.1560754)
Supplement: Supplemental Material [file kgmi-10-04-1560754-s001.docx]

**Supplementary Table 1:** **Median percent abundance (range) for taxa with relative abundances that differed significantly by time alone.** Median (range) percent relative abundances of different taxa in feces collected at baseline (days 5-7), at the conclusion of antibiotic administration (days 26-28), and after a 6 week washout (days 68-70) from 16 healthy cats, 8 per group,^+^ that received 75 mg clindamycin followed 1 hour later by either 2 capsules of placebo or synbiotic PO once daily for 21 days. ^+^Feces not available from 4 cats at time point 26-28 because of early termination of treatment due to severe gastrointestinal signs. Fdr *P*-value = Benjamini and Hochberg False discovery rate (fdr) adjusted *P*-value. Relative abundances that do not share a common superscript letter differed significantly among timepoints based on post-hoc analysis.

|  | **Baseline** | | **Days 26-28** | | **Days 68-70** | | **Fdr P-value** |
| --- | --- | --- | --- | --- | --- | --- | --- |
|  | **Placebo** | **Synbiotic** | **Placebo^+^** | **Synbiotic** | **Placebo** | **Synbiotic** |  |
| ***Actinobacteria*** | 70.73^a^  (26.64-82.04) | 41.74^a^  (6.73-73.17) | 0.91^c^  (0.76-1.20) | 0.93^c^  (0.73-1.09) | 38.59^b^  (18.33-54.74) | 41.13^b^  (20.49-63.38) | <0.01 |
| - *Actinobacteria* | 38.11^a^  (10.88-54.89) | 16.79^a^  (0.71-42.44) | 0.54^b^  (0.45-0.69) | 0.55^b^  (0.48-0.66) | 29.70^a^  (8.01-48.21) | 32.56^a^  (0.65-47.61) | <0.01 |
| - *Actinomycetales* | 0.04^a^  (0.01-0.09) | 0.03^a^  (0.01-0.11) | 0.00^c^  (0.00-0.01) | 0.00^c^  (0.00-0.01) | 0.03^b^  (0.02-0.09) | 0.02^b^  (0.00-0.08) | <0.01 |
| - - *Actinomycetaceae* | 0.03^a^  (0.00-0.09) | 0.03^a^  (0.01-0.09) | 0.00^c^  (0.00-0.01) | 0.00^c^  (0.00-0.01) | 0.02^b^  (0.01-0.08) | 0.01^b^  (0.00-0.06) | <0.01 |
| - - - *Actinomyces* | 0.03^a^  (0.00-0.09) | 0.03^a^  (0.01-0.09) | 0.00^c^  (0.00-0.01) | 0.00^c^  (0.00-0.01) | 0.02^b^  (0.01-0.08) | 0.01^b^  (0.00-0.06) | <0.01 |
| - *Bifidobacteriales* | 38.08^a^  (10.84-54.84) | 16.73^a^  (0.59-42.41) | 0.54^b^  (0.45-0.68) | 0.55^b^  (0.48-0.65) | 29.68^a^  (7.97-48.19) | 32.55^a^  (0.58-47.60) | <0.01 |
| - - *Bifidobacteriaceae* | 38.08^a^  (10.84-54.84) | 16.73^a^  (0.59-42.41) | 0.54^b^  (0.45-0.68) | 0.55^b^  (0.48-0.65) | 29.68^a^  (7.97-48.19) | 32.55^a^  (0.58-47.60) | <0.01 |
| - - - *Bifidobacterium* | 38.06^a^  (10.81-54.83) | 16.72^a^  (0.59-42.38) | 0.54^b^  (0.44-0.68) | 0.55^b^  (0.47-0.65) | 29.66^a^  (7.97-48.19) | 32.54^a^  (0.58-47.60) | <0.01 |
| - *Coriobacteriia* | 26.77^a^  (11.64-40.62) | 24.40^a^  (5.79-30.73) | 0.36^c^  (0.31-0.51) | 0.40^c^  (0.25-0.46) | 8.89^b^  (1.33-18.55) | 16.01^b^  (5.40-19.83) | <0.01 |
| - *Coriobacteriales* | 26.77^a^  (11.64-40.62) | 24.40^a^  (5.79-30.73) | 0.36^c^  (0.31-0.51) | 0.40^c^  (0.25-0.46) | 8.89^b^  (1.33-18.55) | 16.01^b^  (5.40-19.83) | <0.01 |
| - - *Coriobacteriaceae* | 26.77^a^  (11.64-40.62) | 24.40^a^  (5.79-30.73) | 0.36^c^  (0.31-0.51) | 0.40^c^  (0.25-0.46) | 8.89^b^  (1.33-18.55) | 16.01^b^  (5.40-19.83) | <0.01 |
| - - - *___* | 19.59^a^  (0.54-27.70) | 4.97^a^  (0.25-24.95) | 0.16^c^  (0.12-0.29) | 0.17^c^  (0.12-0.25) | 0.41^b^  (0.15-0.47) | 0.37^b^  (0.23-3.75) | <0.01 |
| - - - *Adlercreutzia* | 0.05^a^  (0.01-0.12) | 0.06^a^  (0.03-0.12) | 0.00^c^  (0.00-0.01) | 0.00^c^  (0.00-0.01) | 0.01^b^  (0.00-0.07) | 0.01^b^  (0.00-0.05) | <0.01 |
| - - - *Collinsella* | 10.72^a^  (4.76-14.57) | 8.07^a^  (5.10-22.39) | 0.19^b^  (0.18-0.21) | 0.22^b^  (0.13-0.27) | 8.34^a^  (1.10-17.87) | 13.98^a^  (4.93-18.04) | <0.01 |
| - - - *Slackia* | 0.16^a^  (0.09-0.33) | 0.14^a^  (0.12-1.93) | 0.01^c^  (0.00-0.01) | 0.00^c^  (0.00-0.01) | 0.14^b^  (0.07-0.56) | 0.11^b^  (0.04-1.39) | <0.01 |
| ***Bacteroidetes*** | 0.92^a^  (0.15-3.57) | 1.96^a^  (0.30-9.54) | 0.07^c^  (0.05-0.15) | 0.07^c^  (0.06-0.49) | 0.15^b^  (0.07-3.85) | 1.01^b^  (0.23-3.62) | <0.01 |
| - *Bacteroidia* | 0.92^a^  (0.15-3.57) | 1.96^a^  (0.30-9.54) | 0.07^c^  (0.05-0.15) | 0.07^c^  (0.06-0.49) | 0.15^b^  (0.07-3.85) | 1.01^b^  (0.23-3.62) | <0.01 |
| - *Bacteroidales* | 0.92^a^  (0.15-3.57) | 1.96^a^  (0.30-9.54) | 0.07^c^  (0.05-0.15) | 0.07^c^  (0.06-0.49) | 0.15^b^  (0.07-3.85) | 1.01^b^  (0.23-3.62) | <0.01 |
| - - *Bacteroidaceae* | 0.57^a^  (0.09-3.48) | 1.54^a^  (0.24-7.27) | 0.06^b^  (0.04-0.14) | 0.06^b^  (0.05-0.46) | 0.13^a^  (0.04-2.66) | 0.97^a^  (0.21-3.49) | <0.01 |
| - - - *Bacteroides* | 0.57^a^  (0.09-3.48) | 1.54^a^  (0.24-7.27) | 0.06^b^  (0.04-0.14) | 0.06^b^  (0.05-0.46) | 0.13^a^  (0.04-2.66) | 0.97^a^  (0.21-3.49) | <0.01 |
| - - *Porphyromonadaceae* | 0.02^a^  (0.01-0.17) | 0.23^a^  (0.01-2.15) | 0.00^c^  (0.00-0.01) | 0.01^c^  (0.00-0.02) | 0.01^b^  (0.00-1.13) | 0.04^b^  (0.00-0.09) | <0.01 |
| - - - *Parabacteroides* | 0.02^a^  (0.01-0.17) | 0.23^a^  (0.01-2.15) | 0.00^c^  (0.00-0.01) | 0.01^c^  (0.00-0.02) | 0.01^b^  (0.00-1.13) | 0.04^b^  (0.00-0.09) | <0.01 |
| - - *Prevotellaceae* | 0.08^a^  (0.01-0.36) | 0.07^a^  (0.05-1.51) | 0.01^b^  (0.00-0.01) | 0.01^b^  (0.00-0.01) | 0.01^b^  (0.00-0.05) | 0.01^b^  (0.00-0.05) | <0.01 |
| - - - *Prevotella* | 0.08^a^  (0.01-0.36) | 0.07^a^  (0.05-1.51) | 0.01^b^  (0.00-0.01) | 0.01^b^  (0.00-0.01) | 0.01^b^  (0.00-0.05) | 0.01^b^  (0.00-0.05) | <0.01 |
| ***Firmicutes*** | 25.44^b^  (16.47-71.35) | 55.52^b^  (24.91-84.79) | 90.71^a^  (81.37-97.49) | 88.53^a^  (74.79-96.66) | 59.97^b^  (44.60-77.07) | 56.72^b^  (32.39-78.73) | <0.01 |
| - *Bacilli* | 2.36^c^  (1.22-10.59) | 7.78^c^  (1.91-19.92) | 48.92^a^  (33.22-74.33) | 29.41^a^  (9.88-70.51) | 21.37^b^  (5.87-73.75) | 14.71^b^  (4.40-48.71) | <0.01 |
| - *Bacillales* | 0.02^b^  (0.00-0.07) | 0.04^b^  (0.01-0.07) | 0.09^a^  (0.04-0.17) | 0.07^a^  (0.04-0.16) | 0.04^b^  (0.01-0.06) | 0.04^b^  (0.01-0.07) | <0.01 |
| - *Lactobacillales* | 1.99^c^  (1.08-7.65) | 3.98^c^  (1.71-7.68) | 48.18^a^  (32.72-73.35) | 28.92^a^  (9.59-69.81) | 18.81^b^  (1.57-73.40) | 7.80^b^  (1.26-41.29) | <0.01 |
| - *Clostridia* | 20.32^ab^  (12.98-57.77) | 43.99^ab^  (21.85-73.02) | 24.84^a^  (15.51-50.34) | 51.10^a^  (13.38-68.19) | 27.83^b^  (2.94-54.53) | 23.17^b^  (13.96-69.14) | 0.04 |
| - *Clostridiales* | 20.32^ab^  (12.98-57.77) | 43.99^ab^  (21.85-73.02) | 24.84^a^  (15.51-50.34) | 51.10^a^  (13.38-68.19) | 27.83^b^  (2.94-54.53) | 23.17^b^  (13.96-69.14) | 0.04 |
| - - *Clostridiales*; Other | 0.83^a^  (0.36-1.34) | 0.65^a^  (0.38-1.71) | 0.09^b^  (0.03-0.27) | 0.18^b^  (0.03-0.28) | 0.16^b^  (0.03-0.44) | 0.17^b^  (0.07-0.44) | <0.01 |
| - - - Other | 0.83^a^  (0.36-1.34) | 0.65^a^  (0.38-1.71) | 0.09^b^  (0.03-0.27) | 0.18^b^  (0.03-0.28) | 0.16^b^  (0.03-0.44) | 0.17^b^  (0.07-0.44) | <0.01 |
| - - *Clostridiaceae* | 7.83^b^  (2.48-15.32) | 16.91^b^  (2.61-38.39) | 21.29^a^  (13.32-34.22) | 14.60^a^  (8.71-48.43) | 9.34^b^  (1.29-20.40) | 4.33^b^  (1.30-29.84) | <0.01 |
| - - - Other | 0.73^b^  (0.49-1.10) | 1.46^b^  (0.65-3.12) | 15.88^a^  (7.32-26.38) | 3.93^a^  (1.79-38.25) | 1.07^b^  (0.59-4.94) | 1.19^b^  (0.55-14.00) | <0.01 |
| - - - ___ | 6.76^a^  (1.80-13.41) | 14.66^a^  (1.72-33.56) | 2.92^b^  (1.35-5.89) | 6.41^b^  (1.02-16.50) | 7.87^ab^  (0.47-15.62) | 1.20^ab^  (0.56-14.37) | 0.01 |
| - - - *Clostridium* | 0.17^b^  (0.09-0.29) | 0.31^b^  (0.13-1.96) | 3.04^a^  (1.34-4.09) | 0.90^a^  (0.43-6.69) | 0.22^b^  (0.18-0.71) | 0.27^b^  (0.12-0.94) | <0.01 |
| - - - SMB53 | 0.22^a^  (0.06-0.52) | 0.47^a^  (0.05-1.15) | 0.02^b^  (0.01-0.03) | 0.04^b^  (0.01-0.37) | 0.29^b^  (0.01-0.60) | 0.04^b^  (0.01-0.53) | <0.01 |
| - - *Peptococcaceae* | 0.48^a^  (0.05-1.05) | 1.46^a^  (0.65-3.16) | 0.06^b^  (0.03-0.09) | 0.06^b^  (0.02-0.10) | 0.05^b^  (0.03-0.55) | 0.06^b^  (0.05-0.14) | <0.01 |
| - - - *Peptococcus* | 0.48^a^  (0.05-1.05) | 1.46^a^  (0.65-3.16) | 0.06^b^  (0.03-0.09) | 0.06^b^  (0.02-0.10) | 0.05^b^  (0.03-0.55) | 0.06^b^  (0.05-0.14) | <0.01 |
| - - - *___* | 0.66^a^  (0.13-2.69) | 2.72^a^  (0.75-3.97) | 0.18^b^  (0.10-0.45) | 0.20^b^  (0.10-2.58) | 0.14^b^  (0.10-0.63) | 0.19^b^  (0.14-0.61) | <0.01 |
| - - - *Faecalibacterium* | 0.0114^a^  (0.0000-0.1600) | 0.0700^a^  (0.0029-3.1543) | 0.0043^b^  (0.0029-0.0143) | 0.0057^b^  (0.0000-0.0143) | 0.0000^b^  (0.0000-0.0229) | 0.0057^b^  (0.0000-0.0200) | 0.03 |
| - - - *Ruminococcus* | 0.03^a^  (0.01-0.07) | 0.04^a^  (0.01-0.10) | 0.01^b^  (0.00-0.01) | 0.01^b^  (0.00-0.02) | 0.02^a^  (0.01-0.04) | 0.02^a^  (0.00-0.06) | 0.01 |
| - - *Veillonellaceae* | 1.03^a^  (0.16-3.47) | 1.64^a^  (0.09-2.89) | 0.12^b^  (0.04-0.17) | 0.06^b^  (0.04-0.15) | 0.09^b^  (0.04-0.56) | 0.12^b^  (0.06-1.29) | <0.01 |
| - - - *Megamonas* | 0.04^a^  (0.01-1.14) | 0.04^a^  (0.01-0.07) | 0.02^b^  (0.01-0.05) | 0.01^b^  (0.00-0.04) | 0.02^a^  (0.01-0.42) | 0.03^a^  (0.01-1.17) | 0.01 |
| - - - *Megasphaera* | 0.75^a^  (0.12-3.03) | 1.52^a^  (0.06-2.87) | 0.08^b^  (0.02-0.12) | 0.05^b^  (0.02-0.12) | 0.05^b^  (0.03-0.12) | 0.07^b^  (0.04-0.12) | <0.01 |
| - - - *Phascolarctobacterium* | 0.0171^a^  (0.0000-0.0514) | 0.0129^a^  (0.0029-0.0971) | 0.0057^b^  (0.0000-0.0143) | 0.0057^b^  (0.0000-0.0114) | 0.0029^b^  (0.0000-0.0086) | 0.0029^b^  (0.0000-0.0086) | 0.01 |
| - *Erysipelotrichi* | 0.93^b^  (0.25-20.67) | 2.92^b^  (0.40-7.80) | 9.05^a^  (1.99-19.06) | 7.15^a^  (1.39-18.80) | 3.62^b^  (0.27-13.73) | 1.32^b^  (0.39-10.36) | <0.01 |
| - *Erysipelotrichiales* | 0.93^b^  (0.25-20.67) | 2.92^b^  (0.40-7.80) | 9.05^a^  (1.99-19.06) | 7.15^a^  (1.39-18.80) | 3.62^b^  (0.27-13.73) | 1.32^b^  (0.39-10.36) | <0.01 |
| - - *Erysipelotrichaceae* | 0.93^b^  (0.25-20.67) | 2.92^b^  (0.40-7.80) | 9.05^a^  (1.99-19.06) | 7.15^a^  (1.39-18.80) | 3.62^b^  (0.27-13.73) | 1.32^b^  (0.39-10.36) | <0.01 |
| - - - *___* | 0.17^b^  (0.09-1.15) | 0.31^b^  (0.12-0.79) | 8.52^a^  (1.71-18.17) | 6.65^a^  (1.16-18.07) | 0.35^b^  (0.13-0.87) | 0.31^b^  (0.17-2.91) | <0.01 |
| - - - *[Eubacterium]* | 0.27^a^  (0.10-11.09) | 1.28^a^  (0.21-5.05) | 0.10^b^  (0.09-0.14) | 0.11^b^  (0.07-0.13) | 1.73^a^  (0.07-10.99) | 0.28^a^  (0.10-9.79) | 0.01 |
| ***Proteobacteria*** | 0.57^b^  (0.27-2.69) | 0.78^b^  (0.32-1.73) | 8.45^a^  (1.51-17.35) | 10.33^a^  (2.35-23.76) | 1.60^b^  (0.49-5.98) | 0.55^b^  (0.21-3.07) | <0.01 |
| - *Episilonproteobacteria* | 0.07^a^  (0.01-2.20) | 0.09^a^  (0.03-0.95) | 0.03^b^  (0.00-1.57) | 0.02^b^  (0.01-0.22) | 0.04^b^  (0.00-0.09) | 0.03^b^  (0.02-0.08) | <0.01 |
| - *Campylobacterales* | 0.07^a^  (0.01-2.20) | 0.09^a^  (0.03-0.95) | 0.03^b^  (0.00-1.57) | 0.02^b^  (0.01-0.22) | 0.04^b^  (0.00-0.09) | 0.03^b^  (0.02-0.08) | <0.01 |
| - - *Campylobacteraceae* | 0.01^a^  (0.00-0.08) | 0.03^a^  (0.00-0.33) | 0.00^b^  (0.00-0.01) | 0.00^b^  (0.00-0.01) | 0.00^b^  (0.00-0.01) | 0.00^b^  (0.00-0.01) | 0.02 |
| - *Gammaproteobacteria* | 0.35^c^  (0.22-0.75) | 0.34^c^  (0.20-1.16) | 8.38^a^  (1.46-15.71) | 10.25^a^  (2.12-23.65) | 1.51^b^  (0.40-5.89) | 0.47^b^  (0.17-3.04) | <0.01 |
| - *Enterobacteriales* | 0.25^c^  (0.16-0.65) | 0.27^c^  (0.17-1.15) | 8.33^a^  (0.97-15.66) | 10.23^a^  (1.92-23.60) | 1.46^b^  (0.39-5.88) | 0.38^b^  (0.16-3.03) | <0.01 |
| - - *Enterobacteriaceae* | 0.25^c^  (0.16-0.65) | 0.27^c^  (0.17-1.15) | 8.33^a^  (0.97-15.66) | 10.23^a^  (1.92-23.60) | 1.46^b^  (0.39-5.88) | 0.38^b^  (0.16-3.03) | <0.01 |

**Supplementary Table 2. Fecal metabolite profiles for 100 compounds that differed significantly by time alone in healthy cats administered clindamycin followed 1 hour later by either a placebo or synbiotic.** Median (range) peak height of metabolites in feces collected at baseline (days 5-7), at the conclusion of antibiotic administration (days 26-28), and after a week washout (days 68-70) from 16 healthy cats, 8 per group,^+^ that received 75 mg clindamycin followed 1 hour later by either 2 capsules of placebo or synbiotic PO once daily for 21 days. ^+^Feces not available from 4 cats at time point 26-28 because of early termination of treatment due to severe gastrointestinal signs. Fdr *P*-value = Benjamini and Hochberg False discovery rate (fdr) adjusted *P*-value. Metabolite profiles that do not share a common superscript letter differed significantly among timepoints based on post-hoc analysis.

|  | **Days 5-7 (Baseline)** | | **Days 26-28** | | **Days 68-70** | | **fdr *P*-value** |
| --- | --- | --- | --- | --- | --- | --- | --- |
| **Metabolite** | **Placebo** | **Synbiotic** | **Placebo** | **Synbiotic** | **Placebo** | **Synbiotic** |  |
| 4_hydroxyhippuric_acid | 1,310  (580-6,187)^a^ | 374  (158-8,944)^a^ | 206  (106-1,879)^ab^ | 573  (211-1,813)^ab^ | 222  (53-846)^b^ | 298  (43-1,062)^b^ | 0.05 |
| 1_2_anhydro_myo_inositol | 10,143  (4,125-15,394)^a^ | 2,795  (854-11,787)^a^ | 11,629  (8,732-16,314)^a^ | 7,931  (449-15,429)^a^ | 4,308  (639-12,304)^b^ | 2,807  (1,750-9,445)^b^ | 0.03 |
| 2_monoolein | 3,723  (668-16,348)^a^ | 5,554  (3,572-21,900)^a^ | 7,427  (5,710-12,809)^a^ | 6,951  (3,091-15,437)^a^ | 3,245  (2,203-7,076)^b^ | 2,255  (596-6,912)^b^ | <0.01 |
| 4_hydroxybutyric acid | 2,269  (644-8,014)^a^ | 971  (452-3,697)^a^ | 1,751  (1,199-3,123)^a^ | 1,876  (1,198-2,240)^a^ | 1,041  (343-1,796)^b^ | 905  (273-1,203)^b^ | 0.01 |
| cellobiose | 21,065  (2,701-39,188)^a^ | 4,832  (318-37,705)^a^ | 49,231  (8,116-247,769)^a^ | 5,255  (3,566-11,868)^a^ | 2,774  (823-6,438)^b^ | 3,282  (271-8,657)^b^ | 0.01 |
| galacturonic acid | 9,111  (1,015-20,250)^a^ | 2,422  (996-14,766)^a^ | 14,049  (1,985-20,966)^a^ | 8,317  (1,053-26,505)^a^ | 695  (413-4,454)^b^ | 1,101  (251-9,683)^b^ | <0.01 |
| alanine | 782,140  (384,941-1,085,580)^ab^ | 771,314  (231,203-1,093,786)^ab^ | 303,075  (125,120-401,150)^b^ | 257,468  (174,760-418,633)^b^ | 656,118  (434,687-1,243,494)^a^ | 693,060  (489,700-789,906)^a^ | <0.01 |
| 2,3-dihydroxy-butanoic acid | 289  (21-1,034)^a^ | 175  (52-1,606)^a^ | 82  (59-306)^b^ | 50  (8-226)^b^ | 146  (58-1,832)^a^ | 245  (111-901)^a^ | 0.02 |
| 2,4-diaminobutyric acid | 2,291  (1,388-5,136)^a^ | 1,768  (366-9,508)^a^ | 312  (167-441)^b^ | 323  (172-686)^b^ | 3,413  (266-12,630)^a^ | 2,981  (635-6,901)^a^ | <0.01 |
| 2-ketoisocaproic acid | 3,041  (1,509-7,091)^a^ | 6,235  (2,729-10,036)^a^ | 1,857  (1,216-2,035)^b^ | 2,160  (1,244-2,814)^b^ | 2,671  (2,090-14,394)^a^ | 3,161  (2,257-13,365)^a^ | <0.01 |
| 3_4_dihydroxybenzoic acid | 10,816  (3,732-21,603)^a^ | 4,262  (2,483-20,760)^a^ | 2,831  (2,069-4,290)^b^ | 3,029  (2,092-3,525)^b^ | 7,107  (2,617-33,854)^a^ | 19,317  (1,085-48,873)^a^ | 0.05 |
| 3,4-dihydroxyphenyl-acetic acid | 1,615  (962-2,680)^a^ | 803  (314-3,602)^a^ | 425  (223-724)^b^ | 828  (383-2,525)^b^ | 1,210  (710-9,369)^a^ | 2,352  (180-20,565)^a^ | 0.04 |
| 3_aminoisobutyric acid | 1,567  (832-3,538)^a^ | 5,230  (2,628-6,725)^a^ | 2,696  (89-3,878)^b^ | 923  (107-3,548)^b^ | 5,630  (367-7,288)^a^ | 4,558  (929-7,447)^a^ | 0.02 |
| 3_hydroxy3methylglutaric acid | 1,210  (272-2,505)^a^ | 164  (64-5,028)^a^ | 105  (74-177)^b^ | 139  (86-600)^b^ | 419  (219-1,246)^a^ | 537  (27-2,006)^a^ | 0.02 |
| 4_hydroxybenzoate | 22,974  (15,351-46,996)^a^ | 11,663  (1,061-24,039)^a^ | 4,288  (1,355-11,641)^b^ | 4,153  (1,726-14,652)^b^ | 16,556  (5,239-44,331)^a^ | 19,013  (1,301-41,178)^a^ | 0.02 |
| adenine | 17,905  (7,329-45,632)^a^ | 6,560  (2,489-52,613)^a^ | 4,850  (1,913-8,219)^b^ | 1,850  (1,308-11,043)^b^ | 13,687  (6,454-33,843)^a^ | 12,384  (1,025-32,845)^a^ | <0.01 |
| beta_alanine | 2,741  (1,635-9,396)^a^ | 6,454  (1,803-19,473)^a^ | 343  (206-4,217)^b^ | 1,047  (242-2,850)^b^ | 4,461  (308-20,234)^a^ | 11,807  (408-49,539)^a^ | 0.02 |
| ferulic acid | 6,178  (3,527-18,398)^a^ | 1,634  (486-6,925)^a^ | 465  (312-703)^b^ | 743  (342-51,727)^b^ | 2,584  (722-8,038)^a^ | 2,531  (94-14,464)^a^ | <0.01 |
| fructose | 9,149  (2,507-28,556)^a^ | 5,897  (2,067-20,437)^a^ | 1,241  (582-5,603)^b^ | 1,879  (220-6,215)^b^ | 7,597  (612-31,002)^a^ | 6,250  (631-19,800)^a^ | 0.02 |
| glutamine | 5,407  (3,013-11,710)^a^ | 5,764  (1,623-31,015)^a^ | 1,578  (824-4,090)^b^ | 2,956  (946-5,119)^b^ | 5,668  (491-13,483)^a^ | 3,526  (1,421-9,331)^a^ | 0.01 |
| glutaric acid | 522  (359-856)^a^ | 484  (324-1,791)^a^ | 102  (31-214)^b^ | 174  (60-1,138)^b^ | 326  (68-791)^a^ | 459  (153-1,251)^a^ | 0.02 |
| glycine | 92,756  (58,143-180,584)^a^ | 66,287  (22,418-111,565)^a^ | 26,021  (13,268-36,735)^b^ | 28,150  (19,555-57,764)^b^ | 76,534  (23,280-134,525)^a^ | 88,910  (49,884-120,290)^a^ | <0.01 |
| guanine | 8,991  (5,011-13,767)^a^ | 5,261  (764-13,856)^a^ | 2,371  (1,861-5,182)^b^ | 1,582  (283-5,724)^b^ | 6,610  (4,747-11,063)^a^ | 6,302  (756-14,689)^a^ | <0.01 |
| homoserine | 3,008  (2,105-5,795)^a^ | 2,476  (2,033-4,579)^a^ | 1,476  (857-1,927)^b^ | 949  (781-1,073)^b^ | 2,580  (1,448-4,966)^a^ | 2,778  (1,882-4,668)^a^ | <0.01 |
| hypoxanthine | 29,263  (4,611-80,297)^a^ | 26,072  (11,669-53,041)^a^ | 7,745  (2,035-18,234)^b^ | 9,416  (3,687-16,833)^b^ | 23,115  (11,961-30,095)^a^ | 22,338  (9,838-54,891)^a^ | 0.01 |
| indole_3_lactate | 110,344  (72,690-224,874)^a^ | 134,566  (33,221-453,636)^a^ | 26,768  (13,271-102,119)^b^ | 75,418  (44,683-184,909)^b^ | 117,419  (65,124-188,639)^a^ | 162,374  (1,430-338,952)^a^ | 0.01 |
| isoleucine | 330,838  (146,928-537,925)^a^ | 365,103  (110,720-454,643)^a^ | 120,917  (70,000-154,899)^b^ | 92,525  (23,970-130,426)^b^ | 243,534  (202,229-534,667)^a^ | 382,197  (203,031-491,168)^a^ | <0.01 |
| leucine | 478,561  (262,569-632,516)^a^ | 456,814  (109,184-583,630)^a^ | 209,959  (139,897-307,417)^b^ | 108,699  (66,628-214,707)^b^ | 328,766  (242,027-1,162,806)^a^ | 497,891  (355,841-760,321)^a^ | <0.01 |
| methionine | 65,368  (17,681-84,112)^a^ | 47,392  (11,606-70,473)^a^ | 15,845  (12,394-25,722)^b^ | 19,808  (8,339-23,995)^b^ | 35,082  (20,355-80,215)^a^ | 43,634  (12,060-102,264)^a^ | <0.01 |
| nicotinic acid | 27,392  (8,399-37,799)^a^ | 10,846  (3,291-30,442)^a^ | 8,457  (6,084-12,797)^b^ | 7,885  (5,821-22,912)^b^ | 17,225  (13,161-25,521)^a^ | 16,415  (1,103-34,060)^a^ | 0.03 |
| norvaline | 4,927  (1,270-29,304)^a^ | 3,804  (696-12,135)^a^ | 1,177  (463-1,885)^b^ | 1,012  (289-4,530)^b^ | 7,478  (515-91,032)^a^ | 2,516  (1,205-6,333)^a^ | 0.03 |
| orotic acid | 2,243  (857-7,821)^a^ | 2,529  (326-6,538)^a^ | 189  (103-299)^b^ | 334  (105-966)^b^ | 1,188  (496-4,099)^a^ | 1,416  (225-4,151)^a^ | <0.01 |
| piperidone | 30,449  (5,431-78,729)^a^ | 19,762  (635-111,885)^a^ | 458  (265-2,287)^b^ | 1,555  (277-5,492)^b^ | 27,815  (467-178,299)^a^ | 43,904  (832-111,627)^a^ | <0.01 |
| proline | 104,663  (58,442-253,061)^a^ | 123,218  (21,874-222,734)^a^ | 31,236  (18,656-62,711)^b^ | 29,371  (14,884-57,299)^b^ | 169,027  (36,265-224,277)^a^ | 259,125  (48,288-310,002)^a^ | 0.01 |
| serine | 117,218  (44,398-170,708)^a^ | 81,447  (44,686-149,991)^a^ | 41,781  (29,397-46,028)^b^ | 36,074  (18,952-50,024)^b^ | 83,982  (50,337-122,916)^a^ | 93,030  (62,874-131,009)^a^ | <0.01 |
| urocanic acid | 865  (479-3,818)^a^ | 1,978  (391-2,887)^a^ | 142  (70-303)^b^ | 201  (80-1,673)^b^ | 2,242  (218-4,251)^a^ | 1,875  (182-3,145)^a^ | <0.01 |
| valine | 436,579  (209,002-568,352)^a^ | 429,492  (128,929-580,943)^a^ | 175,332  (133,982-234,967)^b^ | 107,648  (51,862-220,954)^b^ | 349,106  (269,541-801,602)^a^ | 520,987  (293,863-609,491)^a^ | <0.01 |
| 1_hexadecanol | 1,145  (351-1,529)^a^ | 1,945  (1,030-45,417)^a^ | 299  (256-1,714)^b^ | 1,430  (253-35,380)^b^ | 356  (274-751)^b^ | 576  (290-2,373)^b^ | 0.01 |
| citric acid | 1,313  (369-13,473)^a^ | 342  (158-6,213)^a^ | 195  (68-612)^b^ | 391  (187-1,703)^b^ | 130  (56-868)^b^ | 298  (86-702)^b^ | 0.03 |
| glyceric acid | 14,967  (5,258-32,553)^a^ | 9,500  (1,879-2,445,158)^a^ | 6,760  (4,323-9,734)^b^ | 7,224  (2,450-10,033)^b^ | 5,286  (3,494-13,495)^b^ | 5,641  (1,843-12,006)^b^ | 0.03 |
| inositol_4_monophosphate | 657  (201-4,918)^a^ | 775  (163-1,839)^a^ | 246  (146-411)^b^ | 242  (122-658)^b^ | 191  (61-879)^b^ | 413  (110-1,533)^b^ | 0.01 |
| isopentadecanoic acid | 41,428  (25,355-126,295)^a^ | 92,495  (44,950-173,882)^a^ | 30,204  (26,776-49,252)^b^ | 24,276  (10,430-34,491)^b^ | 19,356  (4,918-256,801)^b^ | 14,396  (4,901-116,494)^b^ | <0.01 |
| pentadecanoic acid | 25,635  (17,767-96,730)^a^ | 65,751  (19,963-118,284)^a^ | 15,268  (5,939-17,510)^b^ | 10,376  (9,721-13,001)^b^ | 9,767  (5,710-13,775)^b^ | 12,034  (6,110-46,327)^b^ | <0.01 |
| phosphoethanolamine | 730  (246-1,362)^a^ | 391  (169-805)^a^ | 107  (87-213)^b^ | 141  (84-357)^b^ | 134  (75-508)^b^ | 152  (81-367)^b^ | <0.01 |
| pipecolinic acid | 1,806  (1,212-3,846)^a^ | 4,544  (1,166-12,059)^a^ | 786  (512-1,822)^b^ | 859  (515-3,181)^b^ | 807  (511-3,834)^b^ | 1,221  (728-5,700)^b^ | <0.01 |
| putrescine | 592,861  (188,643-1,084,643)^a^ | 400,585  (7,787-2,741,972)^a^ | 165,171  (66,399-416,065)^b^ | 82,950  (11,840-672,670)^b^ | 192,405  (16,170-725,961)^b^ | 221,571  (49,993-742,246)^b^ | 0.05 |
| ribose | 76,001  (35,761-118,895)^a^ | 60,166  (11,333-105,242)^a^ | 28,445  (23,060-32,999)^b^ | 23,957  (12,289-54,638)^b^ | 42,152  (11,758-88,810)^b^ | 41,142  (2,498-71,574)^b^ | 0.01 |
| 3-hydroxyphenyl-acetic acid | 529  (172-4,109)^a^ | 3,514  (289-18,478)^a^ | 78  (21-138)^c^ | 291  (26-2,221)^c^ | 214  (69-12,535)^b^ | 252  (152-718)^b^ | <0.01 |
| 3,3-hydroxyphenyl propionic acid | 118,316  (55,264-160,927)^a^ | 139,062  (55,351-212,599)^a^ | 132  (92-377)^c^ | 190  (44-1,634)^c^ | 1,238  (188-190,172)^b^ | 948  (170-181,388)^b^ | <0.01 |
| 3-(4-hydroxyphenyl)-propionic acid | 41,265  (24,170-110,451)^a^ | 71,768  (34,626-143,552)^a^ | 4,156  (1,907-10,735)^c^ | 4,981  (1,442-18,082)^c^ | 18,509  (5,641-48,155)^b^ | 17,346  (4,827-33,522)^b^ | <0.01 |
| citrulline | 12,004  (4,842-19,607)^a^ | 12,852  (8,787-23,738)^a^ | 3,291  (2,725-5,575)^c^ | 4,429  (1,400-15,598)^c^ | 7,059  (4,265-14,616)^b^ | 8,599  (2,968-14,245)^b^ | <0.01 |
| deoxycholic acid | 78,243  (838-288,348)^a^ | 38,492  (8,421-922,997)^a^ | 299  (81-1,329)^c^ | 1,358  (202-16,475)^c^ | 8,383  (84-298,628)^b^ | 4,594  (35-976,489)^b^ | <0.01 |
| lysine | 113,544  (53,280-245,005)^a^ | 150,004  (83,591-250,269)^a^ | 30,311  (19,439-47,095)^c^ | 40,669  (11,134-86,528)^c^ | 98,429  (34,120-249,059)^b^ | 54,535  (34,749-213,209)^b^ | <0.01 |
| N_acetyl_D_galactosamine | 35,480  (13,282-51,065)^a^ | 23,547  (6,340-73,010)^a^ | 5,611  (2,099-7,984)^c^ | 5,298  (2,114-30,972)^c^ | 19,787  (7,341-44,224)^b^ | 13,559  (4,536-44,539)^b^ | <0.01 |
| pseudo_uridine | 20,753  (7,529-63,244)^a^ | 14,786  (10,119-78,634)^a^ | 4,154  (2,395-6,613)^c^ | 5,764  (3,972-11,641)^c^ | 9,434  (3,076-25,611)^b^ | 19,054  (8,769-28,400)^b^ | <0.01 |
| uric acid | 1,864  (226-10,572)^a^ | 2,051  (1,057-8,276)^a^ | 554  (114-934)^c^ | 167  (99-1,306)^c^ | 496  (169-1,367)^b^ | 1,056  (155-4,061)^b^ | <0.01 |
| 3,4-dihydroxyhydrocinnaminic acid | 27,522  (764-39,566)^b^ | 14,896  (1,307-39,007)^b^ | 33,604  (11,036-44,143)^ab^ | 38,149  (17,192-78,347)^ab^ | 40,648  (1,836-363,292)^a^ | 148,715  (1,501-640,134)^a^ | 0.04 |
| succinic acid | 48,717  (1,935-390,402)^b^ | 2,100  (747-271,563)^b^ | 195,371  (21,751-795,120)^ab^ | 40,189  (8,920-1,147,221)^ab^ | 218,304  (1,166-674,751)^a^ | 299,338  (2,113-619,092)^a^ | 0.03 |
| 2-hydroxyhexanoic acid | 5,958  (447-42,483)^b^ | 2,111  (350-23,685)^b^ | 27,095  (22,348-43,624)^a^ | 24,704  (17,602-51,851)^a^ | 37,513  (1,681-55,925)^a^ | 8,903  (170-51,591)^a^ | 0.01 |
| 4-aminobutyric acid | 4,798  (2,330-111,922)^b^ | 2,212  (1,625-14,553)^b^ | 40,821  (18,349-113,143)^a^ | 5,644  (3,811-66,681)^a^ | 17,363  (6,165-28,013)^a^ | 12,446  (1,236-57,235)^a^ | 0.01 |
| acetophenone | 2,244  (1,332-2,961)^b^ | 3,176  (1,587-5,245)^b^ | 3,830  (2,898-5,295)^a^ | 4,681  (2,483-5,303)^a^ | 3,406  (1,411-6,387)^a^ | 3,598  (2,855-8,725)^a^ | 0.03 |
| conduritol_beta_epoxide | 400  (52-2,016)^b^ | 199  (66-380)^b^ | 977  (864-2,948)^a^ | 2,442  (71-10,153)^a^ | 896  (55-9,072)^a^ | 540  (20-6,759)^a^ | 0.02 |
| cysteine | 4,274  (2,945-8,963)^b^ | 2,527  (1,602-5,125)^b^ | 10,964  (6,366-15,036)^a^ | 10,514  (5,900-17,287)^a^ | 5,548  (3,820-13,455)^a^ | 8,971  (652-14,184)^a^ | <0.01 |
| lactic acid | 29,969  (4,279-1,912,722)^b^ | 6,457  (3,129-224,210)^b^ | 1,046,986  (897,705-1,199,654)^a^ | 288,190  (19,338-1,514,659)^a^ | 473,391  (4,123-1,692,997)^a^ | 188,366  (2,810-1,067,482)^a^ | 0.01 |
| Propane-1-3-diol | 274  (194-7,900)^b^ | 354  (161-653)^b^ | 4,287  (2,020-4,881)^a^ | 3,278  (1,375-6,740)^a^ | 1,696  (495-16,144)^a^ | 1,375  (367-11,753)^a^ | <0.01 |
| 1_5_anhydroglucitol | 7,071  (3,682-50,668)^b^ | 4,367  (1,703-9,151)^b^ | 15,842  (9,416-60,525)^a^ | 68,651  (15,112-116,831)^a^ | 21,675  (3,075-34,247)^b^ | 9,810  (1,900-300,280)^b^ | 0.03 |
| 1_kestose | 289  (21-1,034)^b^ | 78  (12-257)^b^ | 3,934  (133-59,254)^a^ | 1,770  (238-153,062)^a^ | 94  (31-1,120)^b^ | 110  (48-937)^b^ | 0.01 |
| beta_gentiobiose | 2,524  (977-4,442)^b^ | 994  (239-7,753)^b^ | 12,299  (9,963-17,286)^a^ | 12,308  (5,784-23,039)^a^ | 2,895  (660-8,686)^b^ | 2,677  (203-9,230)^b^ | <0.01 |
| beta_mannosylglycerate | 1,703  (373-4,029)^b^ | 1,310  (646-6,739)^b^ | 6,228  (4,378-12,698)^a^ | 5,148  (3,128-23,835)^a^ | 943  (359-3,784)^b^ | 1,952  (845-7,502)^b^ | <0.01 |
| cerotinic acid | 795  (315-1,684)^b^ | 597  (253-795)^b^ | 1,207  (769-1,453)^a^ | 861  (603-1,515)^a^ | 425  (215-930)^b^ | 572  (410-833)^b^ | 0.01 |
| daidzein | 414  (46-903)^b^ | 110  (40-379)^b^ | 925  (377-1,258)^a^ | 1,017  (266-6,988)^a^ | 330  (135-1,994)^b^ | 256  (11-1,894)^b^ | 0.02 |
| galactinol | 3,599  (2,489-7,624)^b^ | 1,987  (303-6,532)^b^ | 205,923  (65,868-430,786)^a^ | 15,682  (1,904-456,876)^a^ | 1,784  (800-11,980)^b^ | 2,035  (327-4,761)^b^ | <0.01 |
| galactonic acid | 884  (173-1,538)^b^ | 321  (123-1,217)^b^ | 3,459  (789-6,126)^a^ | 1,037  (740-2,521)^a^ | 731  (418-1,370)^b^ | 1,124  (35-1,621)^b^ | 0.03 |
| glycerol_3_galactoside | 7,359  (3,030-32,284)^b^ | 2,676  (657-17,572)^b^ | 69,087  (12,835-143,104)^a^ | 19,870  (6,996-104,583)^a^ | 3,940  (2,148-25,757)^b^ | 2,931  (726-10,544)^b^ | <0.01 |
| isomaltose | 2,598  (500-17,462)^b^ | 258  (112-36,924)^b^ | 299,062  (6,057-396,691)^a^ | 2,792  (1,124-529,690)^a^ | 1,019  (354-23,830)^b^ | 552  (108-3,536)^b^ | <0.01 |
| maleimide | 1,764  (1,172-3,039)^b^ | 1,702  (754-3,946)^b^ | 2,322  (1,802-3,934)^a^ | 3,509  (1,637-7,734)^a^ | 1,892  (918-3,470)^b^ | 2,087  (1,090-3,780)^b^ | 0.05 |
| maltitol | 279  (168-948)^b^ | 111  (40-1,414)^b^ | 2,068  (793-7,544)^a^ | 1,036  (384-2,673)^a^ | 256  (64-740)^b^ | 295  (46-852)^b^ | <0.01 |
| maltotriitol | 272  (33-3,232)^b^ | 38  (26-2,432)^b^ | 2,852  (2,566-3,823)^a^ | 602  (19-4,745)^a^ | 49  (33-965)^b^ | 67  (24-213)^b^ | 0.03 |
| maltotriose | 1,430  (62-4,101)^b^ | 210  (96-10,792)^b^ | 5,179  (2,969-13,705)^a^ | 1,907  (273-16,006)^a^ | 321  (38-3,050)^b^ | 230  (66-1,354)^b^ | 0.01 |
| melibiose | 78  (17-1,045)^b^ | 45  (15-1,161)^b^ | 1,169  (357-5,232)^a^ | 118  (61-2,663)^a^ | 39  (15-1,836)^b^ | 41  (0-172)^b^ | 0.01 |
| oxoproline | 202,876  (136,780-326,329)^b^ | 89,303  (53,235-226,898)^b^ | 343,440  (189,679-727,638)^a^ | 378,795  (216,524-1,182,053)^a^ | 212,517  (40,990-693,484)^b^ | 137,997  (81,090-318,046)^b^ | 0.01 |
| palmitic acid | 91,232  (63,112-173,704)^b^ | 149,053  (113,583-641,946)^b^ | 209,409  (150,366-358,924)^a^ | 211,463  (114,118-302,929)^a^ | 106,114  (54,435-150,102)^b^ | 164,908  (84,709-482,436)^b^ | 0.01 |
| raffinose | 219  (76-1,034)^b^ | 98  (22-365)^b^ | 75,284  (5,095-117,178)^a^ | 887  (143-153,765)^a^ | 82  (16-1,120)^b^ | 341  (44-908)^b^ | <0.01 |
| sorbitol | 7,656  (4,167-13,188)^b^ | 2,383  (1,089-13,972)^b^ | 17,416  (9,156-46,526)^a^ | 15,905  (9,481-36,299)^a^ | 6,794  (2,035-115,689)^b^ | 5,556  (1,333-41,490)^b^ | 0.05 |
| sucrose | 288  (88-732)^b^ | 177  (20-2,207)^b^ | 11,811  (789-15,323)^a^ | 1,371  (433-22,932)^a^ | 291  (14-70,195)^b^ | 202  (14-1,289)^b^ | <0.01 |
| 2_8_dihydroxyquinoline | 1,295  (365-2,440)^b^ | 503  (200-1,314)^b^ | 4,167  (1,159-19,458)^a^ | 4,838  (1,173-11,307)^a^ | 634  (264-1,119)^c^ | 471  (238-643)^c^ | <0.01 |
| adenosine | 540  (89-1,578)^b^ | 256  (99-1,585)^b^ | 1,068  (558-1,692)^a^ | 1,170  (190-9,152)^a^ | 127  (62-483)^c^ | 159  (117-211)^c^ | <0.01 |
| guanosine | 230  (76-1,322)^b^ | 492  (60-1,389)^b^ | 3,002  (1,063-3,973)^a^ | 1,959  (159-2,958)^a^ | 61  (11-98)^c^ | 90  (44-700)^c^ | <0.01 |
| inosine | 391  (115-2,594)^b^ | 885  (130-2,008)^b^ | 3,003  (1,744-6,445)^a^ | 1,245  (344-4,078)^a^ | 67  (49-539)^c^ | 115  (29-727)^c^ | <0.01 |
| lactose | 1,934  (165-5,591)^b^ | 843  (128-7,022)^b^ | 19,307  (1,193-36,222)^a^ | 991  (487-25,149)^a^ | 135  (75-541)^c^ | 131  (36-1,264)^c^ | <0.01 |
| palatinitol | 544  (321-2,729)^b^ | 378  (143-6,772)^b^ | 7,802  (2,879-15,192)^a^ | 3,705  (461-18,076)^a^ | 305  (79-1,402)^c^ | 226  (99-2,374)^c^ | <0.01 |
| 3-phenyllactic acid | 5,318  (523-20,918)^b^ | 1,366  (275-12,731)^b^ | 4,709  (4,529-23,160)^b^ | 4,363  (442-12,880)^b^ | 26,433  (2,799-50,765)^a^ | 16,257  (185-44,885)^a^ | <0.01 |
| 4_methyl_5_thiazoleeth | 8,720  (2,137-11,130)^b^ | 7,587  (1,238-17,067)^b^ | 6,337  (4,223-9,723)^b^ | 6,736  (3,872-15,922)^b^ | 9,835  (6,291-22,908)^a^ | 16,207  (6,925-29,381)^a^ | 0.01 |
| N_acetylornithine | 1,227  (351-2,839)^b^ | 1,344  (456-7,208)^b^ | 339  (240-1,000)^b^ | 1,593  (178-2,788)^b^ | 2,033  (658-5,301)^a^ | 1,998  (696-6,682)^a^ | 0.02 |
| threonine | 73,852  (60,866-122,781)^b^ | 66,251  (21,273-114,094)^b^ | 58,739  (23,264-70,312)^c^ | 26,322  (16,553-66,635)^c^ | 109,099  (65,450-213,970)^a^ | 109,763  (39,842-250,536)^a^ | <0.01 |
| tyrosine | 248,544  (127,581-529,072)^b^ | 248,446  (127,915-386,329)^b^ | 42,503  (16,446-57,851)^c^ | 77,931  (30,466-179,249)^c^ | 384,695  (193,458-557,681)^a^ | 286,133  (128,258-402,890)^a^ | <0.01 |
| 2_5_dihydroxypyrazine | 512  (455-1,018)^c^ | 499  (197-1,405)^c^ | 978  (738-1,386)^a^ | 1,251  (539-2,752)^a^ | 616  (360-1,012)^b^ | 783  (117-1,691)^b^ | <0.01 |
| butyrolactam | 1,448  (742-1,986)^c^ | 1,050  (601-3,613)^c^ | 9,443  (3,680-17,708)^a^ | 3,500  (2,334-9,700)^a^ | 4,312  (2,037-6,093)^b^ | 2,920  (1,233-8,030)^b^ | <0.01 |
| lactamide | 127  (49-1,867)^c^ | 46  (29-376)^c^ | 5,362  (2,180-7,454)^a^ | 773  (119-7,820)^a^ | 663  (77-11,786)^b^ | 320  (20-3,519)^b^ | <0.01 |
| P-hydroxylphenyl-lactic acid | 2,775  (1,076-8,670)^c^ | 712  (332-3,808)^c^ | 3,183  (1,372-5,234)^b^ | 6,196  (2,315-16,911)^b^ | 15,378  (3,039-33,947)^a^ | 10,557  (469-42,710)^a^ | <0.01 |
